# Neural Network Signal Integration from Thermogas-Dynamic Parameter Sensors for Helicopters Turboshaft Engines at Flight Operation Conditions

**DOI:** 10.3390/s24134246

**Published:** 2024-06-29

**Authors:** Serhii Vladov, Lukasz Scislo, Valerii Sokurenko, Oleksandr Muzychuk, Victoria Vysotska, Serhii Osadchy, Anatoliy Sachenko

**Affiliations:** 1 Department of Scientific Work Organization and Gender Issues, Kremenchuk Flight College of Kharkiv National University of Internal Affairs, 17/6 Peremohy Street, 39605 Kremenchuk, Ukraine; serhii.vladov@univd.edu.ua; 2 Faculty of Electrical and Computer Engineering, Cracow University of Technology, Warszawska 24, 31-155 Craków, Poland; 3 Kharkiv National University of Internal Affairs, Ministry of Internal Affairs of Ukraine, 61080 Kharkiv, Ukraine; rector_hnuvs@ukr.net (V.S.); o.muzychuk23@gmail.com (O.M.); 4 Information Systems and Networks Department, Lviv Polytechnic National University, 12 Bandera Street, 79013 Lviv, Ukraine; 5 Institute of Computer Science, Osnabrück University, 1 Friedrich-Janssen-Street, 49076 Osnabrück, Germany; 6 Flight Operation and Flight Safety Department, Flight Academy of the National Aviation University, 1 Chobanu Stepana Street, 25005 Kropyvnytskyi, Ukraine; srg2005@sfa.org.ua; 7 Research Institute for Intelligent Computer Systems, West Ukrainian National University, 11 Lvivska Street, 46009 Ternopil, Ukraine; as@wunu.edu.ua; 8 Department of Teleinformatics, Kazimierz Pulaski University of Radom, 29, Malczewskiego Street, 26-600 Radom, Poland

**Keywords:** sensor, integration, filtration, helicopter turboshaft engine, neural network, error

## Abstract

The article’s main provisions are the development and application of a neural network method for helicopter turboshaft engine thermogas-dynamic parameter integrating signals. This allows you to effectively correct sensor data in real time, ensuring high accuracy and reliability of readings. A neural network has been developed that integrates closed loops for the helicopter turboshaft engine parameters, which are regulated based on the filtering method. This made achieving almost 100% (0.995 or 99.5%) accuracy possible and reduced the loss function to 0.005 (0.5%) after 280 training epochs. An algorithm has been developed for neural network training based on the errors in backpropagation for closed loops, integrating the helicopter turboshaft engine parameters regulated based on the filtering method. It combines increasing the validation set accuracy and controlling overfitting, considering error dynamics, which preserves the model generalization ability. The adaptive training rate improves adaptation to the data changes and training conditions, improving performance. It has been mathematically proven that the helicopter turboshaft engine parameters regulating neural network closed-loop integration using the filtering method, in comparison with traditional filters (median-recursive, recursive and median), significantly improve efficiency. Moreover, that enables reduction of the errors of the 1st and 2nd types: 2.11 times compared to the median-recursive filter, 2.89 times compared to the recursive filter, and 4.18 times compared to the median filter. The achieved results significantly increase the helicopter turboshaft engine sensor readings accuracy (up to 99.5%) and reliability, ensuring aircraft efficient and safe operations thanks to improved filtering methods and neural network data integration. These advances open up new prospects for the aviation industry, improving operational efficiency and overall helicopter flight safety through advanced data processing technologies.

## 1. Introduction and Related Work

Helicopter turboshaft engines (TEs) are complex technical devices requiring continuous monitoring of their condition [1]. Helicopter TEs’ reliable operation depends on the accurate monitoring of thermogas-dynamic parameters, such as temperature and pressure at the engine’s various points, gas–generator rotor rpm and free turbine rotor speed, fuel consumption and others [2]. Modern monitoring and diagnostic systems use many sensors to obtain this data, which makes it possible to detect deviations from the norm and prevent possible malfunctions promptly. However, under flight operating conditions, sensors are exposed to various factors that affect measurement accuracy [3].

The helicopter turboshaft engine sensors must guarantee that data are received with high accuracy to ensure the helicopter’s safe operation. Sensors that measure gas–generator rotor rpm, free turbine rotor speed, and gas temperature in the compressor turbine front are the engine control system’s critical components [4]. In real operating conditions, situations may arise when sensors begin to provide inadequate information due to various factors, such as noise, interference, sensor malfunctions, etc. Even though the sensor is recognized as operational, the information received may be distorted. This can lead to incorrect conclusions about the engine state and, as a result, to erroneous actions by the crew, which are critically dangerous in flight conditions.

Traditional methods for processing sensor signals often face problems associated with noise, sensor failures, and imperfect mathematical models [5,6,7,8]. As a result, a situation may arise when the control system receives distorted data, which leads to incorrect conclusions about the engine state [9,10]. This is especially critical for in-flight operating conditions, where the measurement accuracy can be affected by vibrations, temperature changes and other external factors [11,12].

To solve these problems, data integration methods [13,14] are of particular importance, which makes it possible to combine information from various sensors and systems, the errors impact minimizing and the diagnostic reliability increase. Data integration is based on the algorithms used to process signals, taking into account their relations and correlations [15]. This makes obtaining a more accurate and complete picture of the engine state possible.

The relevance of data integration methods in helicopter TE control and diagnostic systems is due to several key factors. Increasing helicopter TE reliability is a priority task in aviation [16]. Data integration systems make it possible to integrate and jointly process information from multiple sensors, which significantly increases the accuracy and reliability of engine condition diagnostics [17]. Such systems become indispensable in-flight operating conditions, where failures and errors can lead to serious consequences. They provide continuous monitoring and allow you to quickly respond to any changes in engine operation, preventing potential emergencies.

Secondly, data integration methods significantly reduce the influence of external factors, such as vibrations and temperature fluctuations, which can distort the individual sensors’ readings [18]. It is possible to minimize measurement errors and increase the accuracy of the obtained data by taking into account the relations between various parameters and using correction algorithms [19]. This is especially important in helicopter operations, where the environment and flight conditions can vary significantly, affecting sensor performance.

Thirdly, data integration contributes to early fault detection and deviations from the norm [20]. Combining information from various sensors allows potential problems to be detected faster and more accurately, which makes it possible to carry out timely preventive and repair work [21]. This significantly reduces the risk of sudden failure and accidents, increasing overall flight safety. Early fault detection also helps to extend equipment life and reduce operating costs.

Finally, data fusion techniques are crucial for maintenance optimization [22]. Accurately determining the engine components’ conditions allows for effective maintenance planning, minimizing downtime and reducing costs. This enhances the helicopter’s operational performance and mission readiness.

In [23], an aircraft and spacecraft hull structures state acoustic emission diagnostics method was developed using a distributed fibre-optic sensor system. It is shown that such systems can provide vital information for ensuring safety at aerospace facilities. Green’s function for a single sensor is found, and its features under the pulsed influence of acoustic emission signals are researched. The sensor system’s ability to estimate the coordinates and acoustic emission signal parameters has been determined. Experimental research has confirmed the system’s ability to detect radiation signals even under specific interference conditions. A key disadvantage is the difficulty in distributing fibre optic sensors into existing aerospace designs, which may limit their widespread use and require significant modifications during implementation.

In [24], challenges focused on flexibility and scalability are explored when integrating sensors for Industry 4.0 functions into manufacturing systems. These systems use reconfigurable machines with intelligent actuators connected through a single electromechanical interface. An adaptive sensor integration unit architecture is presented that supports interleaved communication protocols and reduces the number of signal lines and the need for protocol conversion units. A prototype system using dynamic partial FPGA reconfiguration has been demonstrated to be effective in an industrial environment. The key disadvantages are the complexity and high cost of developing and implementing a dynamic partial reconfiguration FPGA system and the need for specialized knowledge to operate and maintain it.

The multi-stage supercharging process in [25] is investigated, which is an effective method for improving the engine’s volumetric efficiency at high altitudes, where the intercooler must withstand high thermal loads caused by high levels of supercharging. However, the existing research’s disadvantages on intercooler performance at high altitudes require additional research to optimize its performance in actual flight conditions.

The research [26] aims to modify engines’ advanced knowledge to run on ammonia to promote decarbonization by demonstrating that compression ignition engines can efficiently burn ammonia using spark plugs. The disadvantage is that existing research is limited, and the available experimental data are randomly distributed without careful design, increasing the results’ uncertainty.

Thus, the data integration methods used in monitoring and control systems for the helicopter TE operation are an integral part of modern technology [23,24,25,26] to increase aircraft operation safety and efficiency. The development and implementation of such methods require an interdisciplinary approach and the advanced advances used in the signal processing [27,28], mathematical modelling [29,30] and artificial intelligence [31,32] field. One of these technologies is the neural network approach [33,34], which has several unique advantages over traditional methods [35,36].

Firstly, neural network algorithms can efficiently process large amounts of data and identify complex dependencies between various parameters, which is impossible when using classical signal processing methods. Neural networks can be trained on large amounts of data, which allows them to adapt to changing operating conditions and improve the accuracy of engine prediction [37].

Secondly, neural network models are highly resistant to noise and data failures, which is especially important in flight conditions, where data may be subject to various external influences. Thanks to the ability to self-train and adapt, neural networks can effectively compensate for distortions and provide more accurate diagnostic results [38].

In addition, neural network approaches make it possible to implement more complex and accurate mathematical models that consider nonlinear dependencies between parameters and their time changes. This provides the helicopter TE operational status with a more in-depth analysis and accurate prediction, which increases the helicopter operation reliability and safety [39].

Introducing neural network technology into helicopter TE control and diagnostic systems is a promising development direction that opens up new opportunities for increasing aircraft operations efficiency and reliability.

The proposed method from helicopter TE thermogas-dynamic parameter sensor signals neural network integration represents significant improvements compared to traditional methods due to the dynamic compensation circuit (sensor signals adjustment in real time to improve control system operations) use and a neural network for closed parameter control loop integration. Dynamic compensation with an adaptive noise suppression device effectively filters noise interference while maintaining the helicopter TE parameter essential characteristics sensor signals, improving the control system’s accuracy and reliability.

**A goal of this work is to develop** a method for the helicopter TE thermogas-dynamic parameters integrating signals coming from sensors in real-time and correcting them if they contain noise or distortion to ensure the sensor readings’ accuracy and reliability, despite the possible noise and distortion, to maintain safe and efficient helicopter control. To reach this goal, it is necessary to solve the following tasks:

1. Diagram development for integrating signals from the helicopter TE thermogas-dynamic parameter sensors based on the filtering method.
2. Neural network development to implement a diagram for integrating signals from the helicopter TE thermogas-dynamic parameter sensors using the filtering method.
3. Neural network training algorithm development.
4. Helicopter TE thermogas-dynamic parameter sensor signals’ analysis and preliminary processing.
5. Conduct a computational experiment to solve the filtering sensors signal task of the helicopter TE thermogas-dynamic parameters (using a gas–generator rotor rpm signal example).
6. Evaluate the results obtained effectiveness according to efficiency metrics (efficiency coefficient, quality coefficient, accuracy, recall, precision, F1-score, etc.).
7. The 1st and 2nd errors are calculated and the results obtained are compared with known analogues.

## 2. Materials and Methods

### 2.1. Diagram Development for Integrating Signals from Helicopter TE Thermogas-Dynamic Parameter Sensors Using the Filtering Method

It is known [40,41,42] that the on-board system for monitoring helicopter TE parameters uses the following sensors: D-2M (records the gas–generator rotor rpm), 14 dual thermocouples T-102 (records the gas temperature in front of the compressor turbine), and D-1M (records free turbine rotor speed). These sensors transmit recorded parameters to the onboard instrument panel, providing the pilot with important information about the engine’s condition. However, all of these sensors are subject to noise, which reduces the accuracy and reliability of the recorded data. This requires the neural network aggregation and filtering methods used for signal processing to reduce the noise influence and increase the information reliability displayed on the dashboard.

In the first stage, data reliability is analyzed, which makes it possible to identify and correct anomalies in the data received by helicopter turboshaft engine sensors. According to the helicopter TE sensors readings at times *t*_1_, *t*_2_, …, *t_n_*, a thermogas-dynamic parameter time series is formed:

(1) $$N_{TC}=\left\{n_{TC\;1},n_{TC\;2},\ldots ,\;n_{TC\;n}\right\},\;T_{G}=\left\{T_{G\;1}^{*},T_{G\;2}^{*},\ldots ,\;T_{G\;n}^{*}\right\},\;N_{FT}=\left\{n_{FT\;1},n_{FT\;2},\ldots ,\;n_{FT\;n}\right\},$$

where *n_TC_*_1_, *n_TC_*_2_, …, *n_TC_* _*n*_ is the gas–generator rotor rpm values at times *t*_1_, *t*_2_, …, *t_n_*; $T_{G\;1}^{*}$, $T_{G\;2}^{*}$, …, $T_{G\;n}^{*}$ is the gas temperature in the compressor turbine values front at times *t*_1_, *t*_2_, …, *t_n_*; and *n_FT_*_1_, *n_FT_*_2_, …, *n_FT_* _*n*_ is the free turbine rotor speed values at times *t*_1_, *t*_2_, …, *t_n_*.

The interquartile range (*IQR*) method is used [43] to remove outliers from the helicopter TE thermogas-dynamic parameters data:

(2) $$IQR=Q_{3}-Q_{1},\;BL=Q_{1}-1.5\cdot IQR,\;TL=Q_{1}+1.5\cdot IQR,$$

where *Q*_1_ and *Q*_3_ are the 1st and 3rd quartiles, respectively, *BL* is the lower limit, and *TL* is the upper limit. 

*IQR* is a statistical tool used to identify and remove outliers from a data set, which is especially important when the helicopter TE thermogas-dynamic parameter time series are analysed. *IQR* is defined as the difference between the 3rd *Q*_3_ and 1st *Q*_1_ quartiles, which are the values below which the data lie at 75% and 25%, respectively. The *IQR* calculation allows us to set the *BL* lower and TL upper bounds. Data that falls outside these boundaries are considered outliers, anomalous values that can skew the analysis. Removing outliers helps ensure data reliability and accuracy, which is critical for helicopter health reliable monitoring and diagnosis, preventing errors and improving operational safety.

*Q*_1_ and *Q*_3_ are calculated for each data set to apply *IQR* to eliminate abnormal data preprocessing. Then, values below *BL* or above *TL* are excluded from the analysis. Once outliers are removed and cleaned, further processing and analysis can be carried out. This helps ensure data reliability and accuracy, which is critical for helicopter TE operational status reliable monitoring and diagnostics, preventing errors and increasing operational safety.

The reference range standard is based on the central tendency and variability statistical measures used to determine normal and anomalous values in helicopter TE thermogas-dynamic parameter data sets recorded by sensors in onboard implementation conditions. In particular, it applies the concepts of quartiles, which divide ordered data into four equal parts. The *Q*_1_ 1st quartile and the *Q*_3_ 3rd quartile are the basis for determining the *IQR*, which is the difference between *Q*_3_ and *Q*_1_. This range covers the middle 50% of the data and provides a value spread measure.

To standardize the helicopter TE thermogas-dynamic parameter values, eliminating differences in their ranges and facilitating subsequent analysis, these values are brought to a single scale using normalization:

(3) $$n_{TC}^{norm}\left(t\right)=\frac{n_{TC}\left(t\right)-\mu \left(n_{TC}\right)}{\sigma \left(n_{TC}\right)},T_{G}^{*\;norm}\left(t\right)=\frac{T_{G}^{*}\left(t\right)-\mu \left(T_{G}^{*}\right)}{\sigma \left(T_{G}^{*}\right)},\;n_{FT}^{norm}\left(t\right)=\frac{n_{FT}\left(t\right)-\mu \left(n_{FT}\right)}{\sigma \left(n_{FT}\right)},$$

where *µ*(•) and *σ*(•) are the helicopters TE thermogas-dynamic parameters *n_TC_*, and $T_{G}^{*}$ and *n_FT_* are the average value and standard deviation, respectively.

Data from the helicopter TE thermogas-dynamic parameters that exceed the established *BL* and *TL* are considered anomalies since they demonstrate significant deviations from the expected values. These deviations may indicate serious problems such as faulty hardware malfunctions or sensors malfunctioning. Anomalous data require special attention and analysis, as they can lead to erroneous conclusions about the system state and, as a result, to potentially dangerous situations. Such anomalies’ identification and correction are critical to maintaining reliability and helicopter TE operation safety.

To do this, for each *i*-th helicopter TE thermogas-dynamic parameter *x_i_*(*t*) and each time moment *t*, the anomaly indicator at is calculated as:

(4) $$a_{t}=\left\{\begin{array}{l} 1,\;if\;x_{i}\left(t\right)<BL\;\;or\;\;x_{i}\left(t\right)>TL,\\ 0,\;if\;\;\;\;\;\;\;\;\;\;\;\;\;\;\;\;\;\;\;BL\leq x_{i}\left(t\right)\leq TL, \end{array}\right.$$

Next, the deviation *d_i_*(*t*) is calculated for each *i*-th helicopter TE thermogas-dynamic parameter *x_i_*(*t*) and each time moment *t*:

(5) $$d_{i}\left(t\right)=\frac{x_{i}\left(t\right)-\mu_{i}}{\sigma_{i}}.$$

The value *d_i_*(*t*) shows how many standard deviations the measured value *x_i_*(*t*) differs from the average value *μ_i_*. If the helicopter turboshaft engine thermogas-dynamic parameter (*n_TC_*, $T_{G}^{*}$, *n_FT_*) for any parameter *a_i_*(*t*) = 1, an alarm is generated about a possible sensor malfunction or deviation in the system operation.

**Figure 1:**
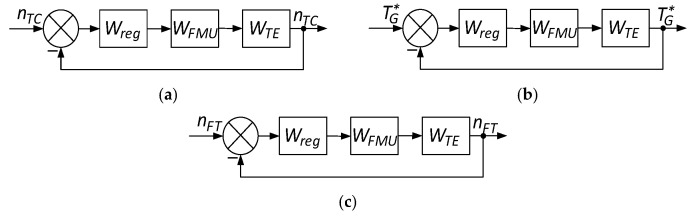
Diagram of closed loops for regulating helicopter turboshaft engine parameters (*W_reg_* is regulator transfer function, *W_FMU_* is fuel dispenser model transfer function, *W_TE_* is helicopter turboshaft engine model transfer function): (**a**) gas–generator rotor rpm, (**b**) gas temperature in front of the compressor turbine, (**c**) free turbine rotor speed (author’s research, based on [44]).

The proposed mathematical model makes it possible to detect sensor data anomalies based on the parameter’s statistical characteristics. Using average values and standard deviations to determine the deviations ensures anomaly detection in the reliable boundaries in the helicopter TE sensors’ functioning. This, in turn, is closely related to the helicopter TE control loops (Figure 1) [44], since accurate sensor data are critical for the engine control systems’ proper functioning. Reliable detection and correction of anomalies ensure the control loops’ correct operation, allowing timely response to changes in engine operation and maintaining optimal operating conditions, which increases overall flight safety and efficiency.

It is worth noting that feedback has been introduced into the control loop (Figure 1), which plays a key role in maintaining the helicopter TE parameters control stability and accuracy. Feedback allows the control system to correct deviations in real time using sensor data. This improves the engine operation reliability and safety, ensuring optimal operating parameters and preventing emergencies.

The helicopter TE parameter regulator in the control loop (Figure 1), connected to the fuel dispenser regulator, acts as a filter. A fuel-metering regulator is present in the control loop to ensure the fuel supply is accurate and has timely regulation, which is necessary to maintain optimal engine operation and quickly respond to changes in operating conditions (Figure 1) [44,45].

**Figure 2:**
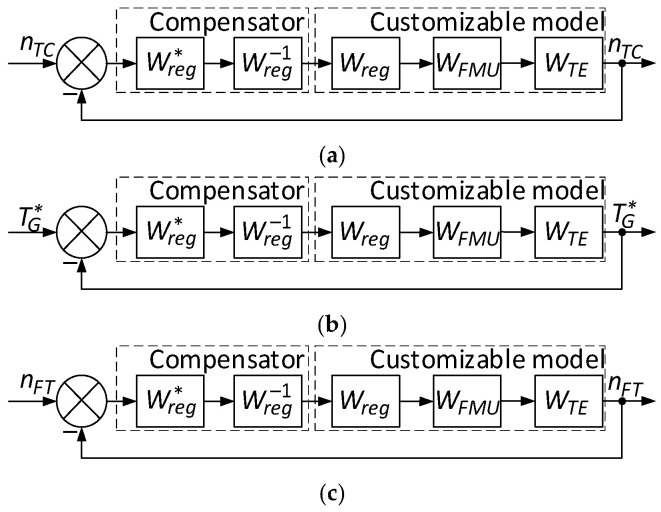
Dynamic compensation diagram in closed loops for regulating helicopter turboshaft engine parameters: (**a**) gas–generator rotor rpm, (**b**) gas temperature in the compressor turbine front, (**c**) free turbine rotor speed (author’s research, based on [44,47,48]).

During the identification process, the closed control loop changes all elements’ parameters in real time (Figure 1). To ensure the desired behaviour, the helicopter TE parameter regulators’ dynamic compensation [46] is carried out by replacing them with a similar structure as the regulators configured in the desired way (Figure 2) [44,47,48].

In Figure 2, the compensator consists of the helicopter TE parameter regulator (*n_TC_*, $T_{G}^{*}$, *n_FT_*) transfer function, with the desired settings $W_{reg}^{*}$ and a transfer function compensating the helicopter TE parameters regulator (*n_TC_*, $T_{G}^{*}$, *n_FT_*) $W_{reg}^{-1}$, and the customized model consists of the transfer functions of the helicopter TE parameters regulator *W_reg_*, the fuel-metering regulator *W_FMU_*, and the engine model.

The dynamic compensation aim is to tune the system to provide the desired system behaviour over the entire operating range and minimize static control error [49]. Transfer functions are used to describe the system. The closed-loop transfer function with a compensator and tunable model can achieve zero static error and optimal control [50,51,52].

The desired system behaviour over the entire operating range is achieved by tuning the controllers. The helicopter TE customizable model structure optimises them for a symmetrical operating mode [47,53]. In this case [47,54], zero static errors are ensured. The transfer function has the form [55] for an open-loop system configured for the symmetrical mode:

(6) $$W^{-1}\left(p\right)=\frac{4\cdot T_{\mu }+1}{8\cdot T_{\mu }^{2}\cdot p^{2}\cdot \left(T_{\mu }\cdot p+1\right)},$$

where *T_μ_* is a small uncompensated time constant, and *p* is the Laplace operator.

During dynamic compensation, the current controller *W_reg_* is replaced by a controller with the desired settings $W_{reg}^{*}$ with the inverse transfer function $W_{reg}^{-1}$ added to compensating the current controller. The closed-loop system overall transfer function, taking into account dynamic compensation, is then determined as *W_closet_*(*p*) = *W_FMU_*(*p*)·*W_TE_*(*p*)·*C*(*p*), where $C\left(p\right)=W_{reg}^{*}\cdot W_{reg}^{-1}$. The Wreg and WFMU controllers are tuned to minimize static errors and provide the required system dynamics to ensure the desired behaviour over the entire operating range.

It is worth considering that various interferences are possible during the helicopter TE operation, which can significantly affect the accuracy of the data received from the sensors. This interference can occur due to external factors, such as electromagnetic fields, vibrations, or sudden changes in the environment, as well as internal factors, including sensor components wear or electronic failures. The interference makes it difficult to correctly read parameters, leading to the engine control circuits’ incorrect operation, increasing the risk of errors in the system operation and reducing the overall helicopter operation reliability and safety. Therefore, the work provides filtering and data processing methods that can effectively eliminate or minimize the influence of interference.

**Figure 3:**
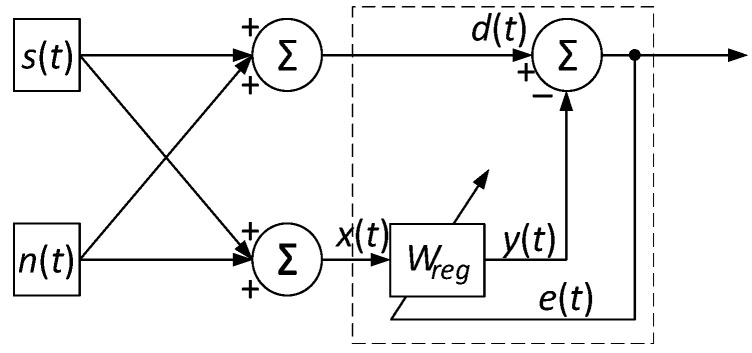
Adaptive device diagram for noise suppression with the helicopter turboshaft engine parameters signal components passage to the reference input (according to B. Widrow and S. Stearns) [56].

In the helicopter TE operation context, where the helicopter TE recorded parameters (*n_TC_*, $T_{G}^{*}$, *n_FT_*) data from sensors’ reliability play a decisive role, it is crucial to consider possible interference that can distort the received signals. To combat this interference, it is effective to use an adaptive interference suppression device, which uses filtering and signal processing methods (Figure 3). This device is highly adaptable to changing operating conditions and helps minimize the interference impact on data quality. Such a device operation involves passing signal components through a reference input, where the signal is compared with a reference value and then corrected or suppressed depending on the deviation degree [56]. This approach allows for effective control and data management, ensuring the helicopter TE control loops reliable operation and increasing overall flight safety.

Figure 3 shows that *s*(*t*) is the useful signal that needs to be restored; *n*_0_(*t*) is the noise added to the useful signal at the main input; *d*(*t*) = *s*(*t*) + *n*_0_(*t*) is the signal at the main input (contaminated with noise); *x*(*t*) is the noise correlated with *n*_0_(*t*) at the reference input (reference input); *y*(*t*) is the output of the adaptive filter, which tries to predict *n*_0_(*t*) based on *x*(*t*); and *e*(*t*) is the error signal. According to Figure 3, it is assumed that the filter has a nonlinearity, which is represented as a function *f*(•) acting on a linear filter output with a transfer function *H*(*p*). Thus, the filter output *y*(*t*) is expressed as:

(7) $$y\left(t\right)=f\left(L^{-1}\left\{H\left(p\right)\cdot X\left(p\right)\right\}\right),$$

where *X*(*p*) is the input signal *x*(*t*) Laplace transform of the helicopter TE-recorded thermogas-dynamic parameters (*n_TC_*, $T_{G}^{*}$, *n_FT_*), and *L*^–1^ is the inverse Laplace transform.

The error signal is defined as the difference between the signal at the main input and the nonlinear filter output, that is:

*e*(*t*) = *d*(*t*) − *y*(*t*). (8)

Nonlinear filter weights’ adaptation is described by a generalized version of the LMS algorithm, which takes nonlinearity into account. It is assumed that **w**(*t*) is the nonlinear filter parameters vector, then the parameter update is defined as:

(9) $$w\left(t+1\right)=w\left(t\right)+\mu \cdot \frac{\partial e\left(t\right)}{\partial w\left(t\right)},$$

where $\frac{\partial e\left(t\right)}{\partial w\left(t\right)}$ is the error signal gradient concerning the filter parameters and *µ* is the training rate.

Models (7)–(9) describe the adaptive noise suppression process using a nonlinear filter and transfer functions, which makes it possible to effectively suppress noise and restore the useful signal *s*(*t*) under complex nonlinear influences.

Models (7)–(9) effectively suppress interference and extract an adaptive system capable of a useful signal *s*(*t*) from the helicopter TE thermogas-dynamic parameters complex nonlinear data characteristic. Equation (7) describes the nonlinear filter output, which consists of two stages: the input signal *X*(*p*), transformed into Laplace space, is passed through a linear filter with a transfer function *H*(*p*). The linear filtering result *f*(•) is subjected to a nonlinear transformation using the function *f*(•). Equation (8) defines the error signal as the difference between the desired signal *d*(*t*) and the nonlinear filter output signal *y*(*t*). Equation (9) describes the nonlinear filter parameter adaptation algorithm based on the modified LMS algorithm.

**Figure 4:**
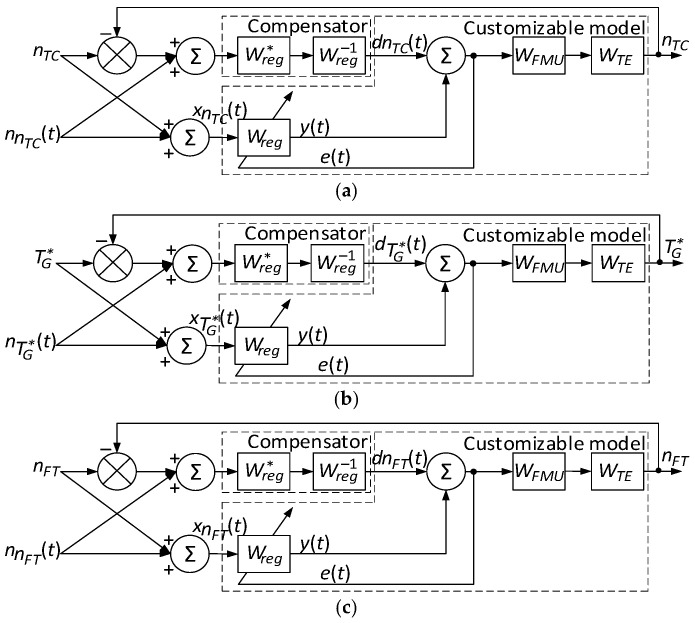
Dynamic compensation diagram in closed loops for regulating the helicopter turboshaft engine parameters with an adaptive noise suppression device with the component signals passage to the reference input: (**a**) gas–generator rotor rpm, (**b**) gas temperature in the compressor turbine front, (**c**) free rotor speed turbines (author’s research).

Thus, the helicopter TE parameters *n_TC_*, $T_{G}^{*}$ and *n_FT_* regulator play the role of an interference suppression device. Therefore, the dynamic compensation circuit in closed loops for regulating helicopter TE parameters takes the form shown in Figure 4. Thus, the registration sensors system *n_TC_*, $T_{G}^{*}$ and *n_FT_*, respectively, include the helicopter TE parameters in three identical control loops (see Figure 4). 

**Figure 5:**
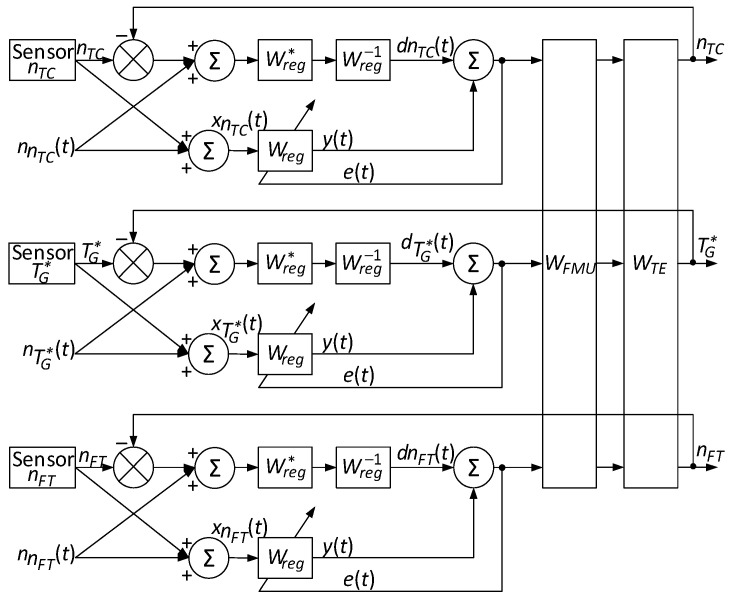
Diagram for integrating closed loops for regulating helicopter turboshaft engine parameters using the filtration method (author’s research).

The transition to a unified integration diagram using the filtering method (Figure 5) makes integrating data from three identical control loops for helicopter TE parameters possible, providing more accurate and reliable control.

This approach allows information from various sensors to be combined into one central control unit, where the data are processed using filtering techniques to eliminate noise and improve measurement accuracy.

Thus, the data integration system provides the helicopter TE parameters with more reliable and accurate control, increasing its performance and durability by optimizing the control systems’ operation. This approach ensures engine stability under various flight modes and external conditions.

The key to the helicopter TE parameters resulting in the control loop (see Figure 5) is the development of helicopter TE parameter regulators that act as adaptive filters. Using a neural network controller, which acts as a filter, is advisable due to its ability to adapt to diverse and dynamic operating conditions [56]. Neural network filters have the unique ability to train from available data and automatically adjust their operation to changes in input signals [57]. This allows them to effectively consider complex nonlinear relationships between parameters and quickly adapt to new conditions without manual reconfiguring. The beneficial qualities mentioned, such as the ability to adapt to diverse and dynamic conditions and automatically adjust to changes in input signals, are characteristic of neural networks in general. However, achieving these benefits depends on the specific implementation and training of the neural network used in this context.

Thus, the key task is the neural network architecture choice, the neural network structure choice, determining the activation functions rules and the neural network hidden layers number, which ensures the engine operational status monitoring with 1st and 2nd type error probability at a minimum level.

### 2.2. Neural Network Architecture Development

**Figure 6:**
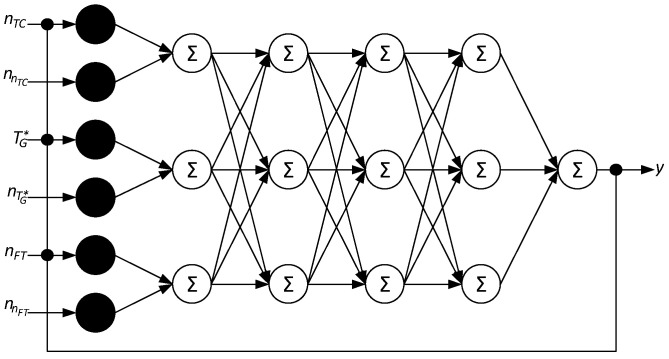
The developed neural network architecture, which implements the closed-loop integration for regulating the helicopter turboshaft engines’ parameters using the filtering method (author’s research).

To solve this task, a multilayer neural network has been developed that processes input data containing useful signals and noise, selects useful signals and provides feedback (Figure 6). The developed neural network input layer contains 6 neurons: 1 is the helicopter TE gas–generator rotor rpm *n_TC_* signal, 2 is the helicopter TE gas–generator rotor rpm *n_TC_* signal interference $n_{n_{TC}}$, 3 is the helicopter TE gas temperature in front of the compressor turbine $T_{G}^{*}$ signal, 4 is the helicopter TE gas temperature in front of the compressor turbine $T_{G}^{*}$ signal interference $n_{T_{G}^{*}}$, 5 is the helicopter TE free turbine rotor speed *n_FT_* signal, and 6 is the helicopter TE free turbine rotor speed *n_FT_* signal interference $n_{n_{FT}}$. In the 1st hidden layer, the parameter signals (inputs 1, 3, 5) are summed with their noise (inputs 2, 4, 6). The 2nd hidden layer means dynamic compensation. The following hidden layers filter the resulting signal, which extracts the useful signal. The output layer contains feedback.

The developed neural network input layer does not perform any data transformation but is intended to receive initial data (helicopter TE parameters *n_TC_*, $T_{G}^{*}$, *n_FT_*) from sensors, which the network’s subsequent layers will process. In this case, 6 input neurons are received, each of input signals, one of which receives *x*_1_ is the helicopter TE gas–generator rotor rpm *n_TC_* signal, *x*_2_ is the helicopter TE gas–generator rotor rpm *n_TC_* signal interference $n_{n_{TC}}$, *x*_3_ is the helicopter TE gas temperature in the compressor turbine $T_{G}^{*}$ signal front, *x*_4_ is the helicopter TE gas temperature in the compressor turbine $T_{G}^{*}$ signal interference front $n_{T_{G}^{*}}$, *x*_5_ is the helicopter TE free turbine rotor speed *n_FT_* signal, and *x*_6_ is the helicopter TE free turbine rotor speed *n_FT_* signal interference $n_{n_{FT}}$. Each input neuron receives one of these signals unchanged, that is, *y_i_* = *x_i_*.

The neural network’s 1st hidden layer operates by summing the signals with their corresponding noise. Each neuron in this layer processes a signal–noise pair (the signal-noise is formed by adding random noise to the pure signal, which corresponds to the actual helicopter flight conditions). This is necessary to prepare the data for subsequent dynamic compensation and filtering. The combined signals allow the model to be trained more efficiently to remove noise and highlight useful data. Thus,

(10) $$h_{1}=x_{1}+x_{2},\;h_{2}=x_{3}+x_{4},\;h_{3}=x_{5}+x_{6},$$

where *h*_1_, *h*_2_, *h*_3_ are the first hidden layer neurons outputs.

Based on the above, the neural network’s 1st hidden layer has three neurons. This layer passes through the neuron without activation since simple summation is required.

The 2nd hidden layer performs the dynamic compensation task. This layer corrects signals to compensate for noise and dynamic changes to improve the data quality before filtering it in subsequent layers. In this layer, neurons are trained to adjust signals taking into account their dynamics and noise. This is achieved by applying trainable weights and activation functions to the summed signals, that is:

(11) $$z_{1}=f\left(w_{11}\cdot h_{1}+w_{12}\cdot h_{2}+w_{13}\cdot h_{3}+b_{1}\right),\;z_{2}=f\left(w_{21}\cdot h_{1}+w_{22}\cdot h_{2}+w_{23}\cdot h_{3}+b_{2}\right),\;z_{3}=f\left(w_{31}\cdot h_{1}+w_{32}\cdot h_{2}+w_{33}\cdot h_{3}+b_{3}\right),$$

where *z*_1_, *z*_2_, and *z*_3_ are the second hidden layer neuron outputs, *w_ij_* are the weights trained during the network training process, *b_i_* are the biases trained during the network training process, and *f*(•) is the activation function.

For the 2nd hidden layer, selecting the ReLU (Rectified Linear Unit) activation function is advisable because ReLU effectively copes with dynamic changes in signals and noise due to its ability to pass positive values unchanged and to null out negative ones. This allows neurons to adapt to different levels of input signals and quickly adjust for dynamic changes, which is important for effective noise compensation. In addition, ReLU helps avoid the gradient fading problem, improving the deep networks training and providing more stable and faster model convergence, which is critical for tasks that require accurate dynamic compensation.

**Note 1.** A critical drawback of the ReLU activation function is the problem of “dying” neurons when input values are negative and the outputs become zero. In this case, neurons stop participating in training since their weight gradient becomes zero. This can cause a significant number of neurons in the network to remain inactive, reducing the model’s overall training ability and degrading its performance. Later in the work, this problem will be solved by modifying the ReLU function.

The 3rd hidden layer (Filtering Layer 1) performs the resulting signals after dynamic compensation filtering in the 1st stage. This layer is designed to reduce noise further and improve the desired signal quality. The 3rd hidden layer applies trainable weights and activation functions to the input signals to filter them and extract useful information. Mathematically, the neural network’s 3rd hidden layer is represented as follows:

(12) $$f_{1}=g\left(w_{11}\cdot z_{1}+w_{12}\cdot z_{2}+w_{13}\cdot z_{3}+b_{1}\right),\;f_{2}=g\left(w_{21}\cdot z_{1}+w_{22}\cdot z_{2}+w_{23}\cdot z_{3}+b_{2}\right),\;f_{3}=g\left(w_{31}\cdot z_{1}+w_{32}\cdot z_{2}+w_{33}\cdot z_{3}+b_{3}\right),$$

where *f*_1_, *f*_2_, and *f*_3_ are the 3rd hidden layer neuron outputs, *w_ij_* is the trainable weights applied to the input signals, *b_i_* is the trainable biases, and *g*(•) is the activation function that helps neurons process input signals nonlinearly, which improves the ability models to isolate useful signals and eliminate noise.

For the 3rd hidden layer, choosing the ReLU activation function is advisable because it can handle noise and extract useful signals efficiently. ReLU allows neurons to only pass positive values through while nulling out negative ones, which helps eliminate unnecessary noise and improves overall signal quality. In addition, ReLU speeds up training by eliminating the gradient decay issues associated with other activation functions and allows the network to better model complex dependencies in data. This makes ReLU ideal for the 1st stage of filtering, effectively extracting useful information from the corrected signals. 

The 4th hidden layer represents the resulting signals filtering the 2nd stage, which follows the filtering 1st stage in the 3rd hidden layer. This layer further improves the wanted signal quality by suppressing the remaining noise and making the signal more distinguishable from the background. The 4th hidden layer also applies trainable weights and activation functions to the input signals to further filter the data and improve the desired signal quality. Mathematically, the fourth neural network hidden layer is represented as follows:

(13) $$g_{1}=h\left(w_{11}\cdot f_{1}+w_{12}\cdot f_{2}+w_{13}\cdot f_{3}+b_{1}\right),\;g_{2}=h\left(w_{21}\cdot f_{1}+w_{22}\cdot f_{2}+w_{23}\cdot f_{3}+b_{2}\right),\;g_{3}=h\left(w_{31}\cdot f_{1}+w_{32}\cdot f_{2}+w_{33}\cdot f_{3}+b_{3}\right),$$

where *g*_1_, *g*_2_, and *g*_3_ are the 4th hidden layer neuron outputs, *w_ij_* is the trainable weights applied to the input signals, *b_i_* is the trainable biases, and *h*(•) is the activation function.

For the 4th hidden layer, choosing the ReLU activation function is also advisable due to its ability to effectively suppress unnecessary negative values, thereby reducing the noise impact and preserving the signal’s positive aspects. This allows the network to more efficiently identify and store useful features present in the data, which is important for the task of integrating and improving signal quality.

Thus, the second, third and fourth hidden layers each have three neurons.

The neural network output layer predicts or classifies the input data according to the given task. In this context, the output layer will predict the system parameters or characteristics based on the input signals after they have been processed and filtered through hidden layers. Given the feedback presence, this layer must also take into account the error received in the previous stages and adjust the network outputs according to this error. For the output neuron, the expression is valid:

(14) $$y=r\left(v_{1}\cdot g_{1}+v_{2}\cdot g_{2}+v_{3}\cdot g_{3}+c\right),$$

where *y* is the helicopter TE parameter (*n_TC_*, $T_{G}^{*}$ and *n_FT_*) predicted output, *v_i_* is the output layer trainable weights, *c* is the trainable bias (bias), and *r*(•) is the activation function.

Taking into account feedback, the error *E* at the network output can be defined as the difference between the predicted output *y* and the expected output *d*, that is:

*E* = *y* − *d*. (15)

The backpropagation algorithm is applied to update the weights $v_{i}$ and bias c of the output layer. The weights and bias are adjusted in the opposite direction to the gradient of the loss function to these parameters. Thus, updating the weights $v_{i}$ and bias c of the output layer will occur according to gradient descent as:

(16) $$∆v_{i}=\alpha \cdot \frac{\partial E}{\partial v_{i}},\;∆c=\alpha \cdot \frac{\partial E}{\partial c},$$

where α is the adaptive training rate, which includes an adaptive change in the training rate depending on the current gradient and the weight updates history, which, according to the AdaGrad algorithm (Adaptive Gradient Algorithm), is defined as:

(17) $$\alpha_{t}=\frac{\eta }{\sqrt{G_{t}+\epsilon }},$$

where *ϵ* is a small constant added for numerical stability (*ϵ* ≈ 10^–8^ is assumed), *α_t_* is the training rate at time *t*, *η* is the initial training rate, and *G_t_* is the accumulated squared gradients up to time *t*, which are updated at each training iteration in the following way:

(18) $$G_{t}=G_{t-1}+{\left(\nabla E\right)}^{2},$$

where ∇*E* is the loss function gradient over the model parameters.

In a developed neural network where the main task is to predict the parameters or characteristics of the system based on the processed data, the use of a linear activation function at the output layer may be appropriate. This is especially relevant since the output values will be helicopter TE parameters *n_TC_*, $T_{G}^{*}$, and *n_FT_* continuous numerical values. A linear activation function will allow the neural network to flexibly adapt to different ranges of output parameter values without limiting them to any specific range.

### 2.3. The ReLU Activation Function Modification

The work proposes to use the innovative activation function Smooth ReLU, developed by this author’s team, which is a ReLU function derivative. The main aim of the ReLU function modification is to create a smoother and more continuous activation function to improve the convergence process and training stability. The proposed modification can significantly affect the neural network efficiency, especially in deep training problems where stability and convergence speed play a key role. The expression describes the Smooth ReLU activation function:

(19) $$f\left(x\right)=\left\{\begin{array}{l} x,\;\;\;\;\;\;\;\;\;\;\;\mathrm{if}\;x>0,\\ \frac{1}{1+e^{-\gamma \cdot x}},\;\mathrm{if}\;x\leq 0, \end{array}\right.$$

where *γ* is a parameter that determines the function “level of smoothness”. For *x* > 0, the function behaves similarly to a regular ReLU, and for *x* ≤ 0 it smoothly transitions to negative values using a sigmoid function. This avoids sudden gradient changes and can speed up neural network training. The proposed Smooth ReLU activation function retains the ReLU benefits, such as no gradient for positive values, while adding smoothness for negative values. This can sometimes improve training, allowing for more stable and faster convergence.

**Theorem** **1.**  *The Smooth ReLU function is continuous over the entire domain of definition*.

**Proof of Theorem 1.**  Let *f*(*x*) be the Smooth ReLU activation function defined according to (18). The Smooth ReLU activation function is continuous for *x* > 0 and *x* < 0, since for *x* > 0 the function *f*(*x*) = *x* is a linear function that is continuous over the definition of the entire domain, and for *x* < 0 $f\left(x\right)=\frac{1}{1+e^{-\gamma \cdot x}}$, which means a modified sigmoid function is continuous throughout the entire domain of the definition.

To prove continuity at *x* = 0, it is necessary to show that the limit of *f*(*x*) as *x* tends to zero from the left is equal to the limit of *f*(*x*) as *x* tends to zero from the right, and that this limit is equal to the value of the function at *x* = 0. Consider the limit:

(20) $$\underset{x\to 0^{-}}{\lim}f\left(x\right)=\underset{x\to 0^{-}}{\lim}\frac{1}{1+e^{-\gamma \cdot x}},$$

in which as x → 0^–^, *γ*·*x* → 0, *e*^–*γ*·*x*^ = 1, thus:

(21) $$\underset{x\to 0^{-}}{\lim}\frac{1}{1+e^{-\gamma \cdot x}}=\frac{1}{1+1}=\frac{1}{2}.$$

Consider the limit:

(22) $$\underset{x\to 0^{+}}{\lim}f\left(x\right)=\underset{x\to 0^{+}}{\lim}x=0.$$

Since the different sides’ limits do not coincide, checking the function value at point x = 0 is necessary. Let us take the value *x* ≠ 0 at point *x* = 0, for example, $f\left(0\right)=\frac{1}{2}$. Next, continuity is revised with the set value $f\left(0\right)=\frac{1}{2}$, that is:

(23) $$\underset{x\to 0^{-}}{\lim}f\left(x\right)=\frac{1}{2},\;\underset{x\to 0^{+}}{\lim}f\left(x\right)=0\neq \frac{1}{2}.$$

Thus, the original function was not continuous at *x* = 0 with the given condition, and an adjustment is required to determine continuity correctly. Then, the Smooth ReLU function is described by the expression:

(24) $$f\left(x\right)=\left\{\begin{array}{l} x,\;\;\;\;\;\;\;\;\;\;\;\;\;\;\;\;\;\mathrm{if}\;x>0,\\ 0\;\;\;\;\;\;\;\;\;\;\;\;\;\;\;\;\;\;\;\mathrm{if}\;x=0,\\ \frac{1}{1+e^{-\gamma \cdot x}},\;\;\;\;\;\;\;\mathrm{if}\;x<0. \end{array}\right.$$

This function, similar to (19), is continuous for *x* > 0 and *x* < 0, and continuity at point *x* = 0 is defined as:

(25) $$\underset{x\to 0^{-}}{\lim}f\left(x\right)=\underset{x\to 0^{-}}{\lim}\frac{1}{1+e^{-\gamma \cdot x}}=\frac{1}{2},\;\underset{x\to 0^{+}}{\lim}f\left(x\right)=0.$$

And its value at point *x* = 0 is zero.

Thus, the adjustment assumes that the function is not smooth at point 0, but remains continuous throughout the definition of the entire domain. The theorem is proven: the function *f*(*x*), defined with correction in the form (24), is continuous over the entire domain of the definition. □

**Figure 7:**
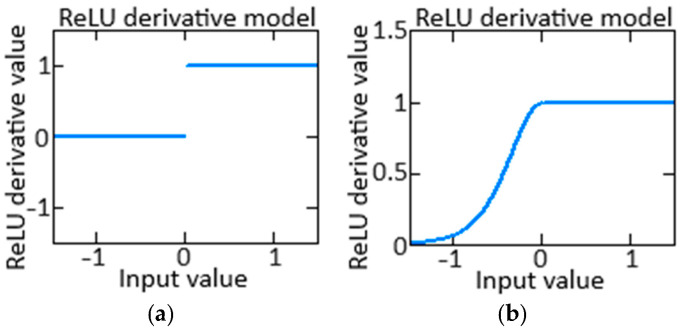
Derivative ReLU functions diagrams: (**a**) traditional ReLU max(0, *x*); (**b**) proposed Smooth ReLU with adjustment (22) (author’s research).

To research the neuron’s activation functions, it is imperative to analyze their derivatives. The activation function derivative allows us to estimate this function change rate in response to changes in input data. In turn, the updating neuron weights process helps optimize during neural network training. The traditional neuron activation function ReLU *f*(*x*) = max(0, *x*) derivative (Figure 7a) has the form:

(26) $$f\prime \left(x\right)=\left\{\begin{array}{c} 1,\;\mathrm{if}\;x>0,\\ 0,\;\mathrm{if}\;x\leq 0, \end{array}\right.$$

The proposed Smooth ReLU neuron activation function with correction (24) derivative (Figure 7b) has the form:

(27) $$f\prime \left(x\right)=\left\{\begin{array}{l} 1,\;\;\;\;\;\;\;\;\;\;\;\;\;\;\;\;\;\mathrm{if}\;x>0,\\ 0,\;\;\;\;\;\;\;\;\;\;\;\;\;\;\;\;\;\mathrm{if}\;x=0,\\ \frac{\gamma \cdot e^{-\gamma \cdot x}}{{\left(1+e^{-\gamma \cdot x}\right)}^{2}},\;\;\;\mathrm{if}\;x<0. \end{array}\right.$$

As can be seen from (26), (27), and Figure 7, the problem with the traditional ReLU function *f*(*x*) = max(0, *x*) is that its derivative is zero for all *x* ≤ 0. This can lead to “dead neurons” in the neural network when neurons stop updating due to the lack of gradient. The advantage of Smooth ReLU is that it always has a non-zero gradient for all values of *x*, including negative ones (except for *x* = 0). This avoids the problem of “dead neurons” and ensures more stable neural network training.

Thus, the proposed Smooth ReLU use with adjustment (24) is mathematically justified since it provides a smooth and continuous gradient throughout the definition domain, which can help improve the convergence and training efficiency of the model.

### 2.4. A Neural Network Training Algorithm Development

The work proposes an algorithm for training a neural network (Figure 6), consisting of the following stages:

1. Weights initialization is initially setting the neural network weight values before starting training. Proper weight initialization is important for efficient and fast network training, as it helps avoid convergence problems and helps achieve more accurate results.

For the developed neural network that uses Smooth ReLU in the hidden layers, it is appropriate to use the He initialization method [58]. The 1st hidden layer weight initialization is not required since this layer performs simple summation. The neural network remaining layers’ weight initialization is carried out as follows:

(28) $$W\sim N\left(0,\frac{2}{n_{i}}\right),$$

where *N*(*μ*, *σ*^2^) is a normal distribution with mean *μ* and variance *σ*^2^, and *n_i_* is the neuron number in the neural network *i*-th layer.

2. Forward propagation is passing the neural network input data through all layers to the output to obtain predictions, in which the input signals’ weighted sums are calculated at each layer, to which an activation function is then applied. This process ensures that the input data are transformed into network output values. Direct propagation is carried out according to (10)–(15).

3. Backpropagation is a neural network training to minimize prediction error by propagating the network output back error calculated through the layers, updating the network’s weights and biases. The main purpose of backpropagation is to adjust the weights in such a way as to reduce the predicted and actual values difference between them. For each layer, the error function gradient concerning its weights and biases is calculated as:

(29) $$\delta_{out}=\frac{\partial E}{\partial Y}=y-d,\;\frac{\partial E}{\partial v_{i}}=\delta_{out}\cdot g_{i},\;\frac{\partial E}{\partial c}=\delta_{out}.$$

For the remaining layers (1st–4th hidden layers) of the neural network, similar calculations are given in Table 1.

4. Updating weights and biases is the neural network’s weights and biases changing process over time to the loss function minimization. The squared gradients accumulation is defined as:

(30) $$G_{w_{ij}^{\left(m\right)}}=G_{w_{ij}^{\left(m\right)}}+{\left(\frac{\partial E}{\partial w_{ij}^{\left(m\right)}}\right)}^{2},$$

where *m* = 2…4 is the neural network hidden layers number (2nd, 3rd and 4th hidden layers, respectively).

Weights and biases are updated according to the expressions:

(31) $$w_{ij}^{\left(m\right)}=w_{ij}^{\left(m\right)}-\frac{\eta }{\sqrt{G_{w_{ij}^{\left(m\right)}}+\epsilon }}\cdot \frac{\partial E}{\partial w_{ij}^{\left(m\right)}},$$

(32) $$c=c-\frac{\eta }{\sqrt{G_{c}+\epsilon }}\cdot \frac{\partial E}{\partial c}.$$

The weights and biases forward propagation, backpropagation, and updating are repeated for all training examples and throughout all training epochs until the convergence criterion is reached.

For the task posed in the work of filtering interference and integrating signals from the helicopter TE thermogas-dynamic parameter sensors using the developed neural network (Figure 6), which implements a diagram for the regulating helicopter TE parameters integrating closed loops using the filtering method (Figure 5), the stopping criterion is a balanced criterion for improving accuracy on the validation set and controlling the neural network retraining.

To create a balanced convergence criterion that takes into account both the improvement in accuracy on the validation set and control for overfitting, a weighted average between the two criteria can be used:

(33) $$C=\alpha \cdot \varepsilon +\left(1-\alpha \right)\cdot O,$$

where *ε* is the accuracy (increase in model accuracy on the validation set (for example, the percentage of correct predictions), *O* is the measure of model retraining control (ratio of error on the validation set to error on the training set), and 0 ≤ *α* ≤ 1 is the coefficient reflecting the increasing accuracy importance compared to the overfitting control.

**Note 2.** An appropriate value for *α* can be chosen depending on the specific task requirements and preferences. For example, if increasing accuracy is more important than controlling for overfitting, then α might be chosen closer to 1. If controlling for overfitting is more important, then α might be chosen closer to 0.

The scientific novelty of the proposed neural network training algorithm lies in the integration of two convergence criteria: increasing accuracy on the validation set and controlling overfitting into a single criterion that allows for balancing, improving model accuracy, and preventing overfitting. The difference from the traditional backpropagation algorithm is that the proposed method not only minimizes the loss function but also takes into account the error dynamics on the validation sample, which allows for maintaining the model generalization ability when achieving a certain level of accuracy. Additionally, the adaptive training rate complements this approach by allowing the model to efficiently and quickly adapt to changes in data and training conditions, which improves its performance in practice.

## 3. Case Study

### 3.1. Analysis and Preliminary Processing Results for Initial Signals from Helicopter TE Thermogas-Dynamic Parameter Sensors

To conduct a computational experiment, data were obtained on the TV3-117 TE thermogas-dynamic parameters [59,60,61], recorded on board the Mi-8MTV helicopter during flight: gas–generator rotor rpm *n_TC_* at times *t*_1_…*t_n_*; gas temperature in front of the compressor turbine $T_{G}^{*}$ at times *t*_1_…*t_n_*; and free turbine rotor speed *n_FT_* at times *t*_1_…*t_n_*. The *n_TC_*, $T_{G}^{*}$, and *n_FT_* values are given in absolute values (Table 2, Table 3 and Table 4).

At the helicopter TE thermogas-dynamic parameter (*n_TC_*, $T_{G}^{*}$ and *n_FT_*) values preliminary processing in the 1st stage, the homogeneity of the training samples is assessed (Table 2, Table 3 and Table 4). According to [59,60,61], the criterion by which the training sample homogeneity is determined is the Fisher–Pearson criterion, which is defined as [62]:

(34) $$\chi^{2}=\frac{\sqrt{N\cdot \left(N-1\right)}}{N-2}\cdot \frac{\frac{1}{N}\cdot \sum_{i=1}^{N}{\left(x_{i}-\bar{x}\right)}^{3}}{{\left(\frac{1}{N}\cdot \sum_{i=1}^{N}{\left(x_{i}-\bar{x}\right)}^{2}\right)}^{\frac{3}{2}}},$$

where *N* = 256 is the training sample size, *x_i_* is the training sample (Table 2, Table 3 and Table 4) *i*-th element value, and $\bar{x}=\frac{1}{n}\cdot \sum_{i=1}^{n}x_{i}$ is the training sample average value.

The significance level adopted in the work is 0.01, which means the probability of introducing a type I error (erroneously rejecting the true null hypothesis) is 1%. That is, while a statistical test indicates a significant result, there is only a 1% chance that the result is due to chance and is due to noise or random variations in the data. This significance indicates strict requirements for the results’ reliability, which is especially important in the context of helicopter TE thermogas-dynamic parameter sensor readings’ accuracy and reliability. The freedom degrees number is 1 (one type of parameter in each training sample: *n_TC_*, $T_{G}^{*}$ or *n_FT_*). Thus, the Fisher–Pearson test’s critical value for one degree of freedom at a significance level of 0.01 was 6.6. The Fisher–Pearson criterion obtained values are $\chi_{n_{TC}}^{2}=4.727$, $\chi_{T_{G}^{*}}^{2}=4.645$, and $\chi_{n_{FT}}^{2}=5.619$ are less than the critical value $\chi_{critical}^{2}=6.6$, which indicates the helicopter TE thermogas-dynamic parameter training sample homogeneity.

To confirm the training sample (see Table 2, Table 3 and Table 4) homogeneity assessing results using the Fisher–Pearson criterion according to [59,60,61], an identical experiment was carried out using the Fisher–Snedecor criterion, which is defined as [63]:

(35) $$F=\frac{S_{1}^{2}}{S_{2}^{2}}=\frac{\frac{1}{n_{1}-1}\cdot \sum_{i=1}^{n_{1}}{\left(x_{i}^{\left(1\right)}-{\bar{x}}^{\left(1\right)}\right)}^{2}}{\frac{1}{n_{2}-1}\cdot \sum_{i=1}^{n_{2}}{\left(x_{i}^{\left(2\right)}-{\bar{x}}^{\left(2\right)}\right)}^{2}},$$

where *n*_1_ and *n*_2_ are the 1st and 2nd training sample sizes, $x_{i}^{\left(1\right)}$ and $x_{i}^{\left(2\right)}$ are the *i*-th element of the 1st and 2nd training samples, respectively, and ${\bar{x}}^{\left(1\right)}$ and ${\bar{x}}^{\left(2\right)}$ are the average values of the 1st and 2nd training samples, respectively.

To calculate the Fisher–Snedecor criterion according to (35), the helicopter TE thermogas-dynamic parameter (see Table 2, Table 3 and Table 4) value training samples, consisting of 256 elements each, are randomly divided into two equal samples of 128 elements each, that is, *n*_1_ = *n*_2_ = 128. The significance level for the Fisher–Snedecor criterion is also accepted as 0.01, and the freedom degrees number is 1, which indicates the helicopter TE parameters one type in each training sample: *n_TC_*, $T_{G}^{*}$ or *n_FT_*. Thus, the Fisher–Snedecor test critical value for one freedom degree at a significance level of 0.01 was 6.6. The Fisher–Snedecor criterion obtained values are $F_{n_{TC}}=4.727$, $F_{T_{G}^{*}}=4.645$, and $F_{n_{FT}}=5.619$ are less than the critical value $F_{critical}=6.6$, which confirms the helicopter TE thermogas-dynamic parameter training sample homogeneity.

At the helicopter TE thermogas-dynamic parameter (*n_TC_*, $T_{G}^{*}$ and *n_FT_*) values preliminary processing in the 1st stage according to [59,60,61], the training and test samples representativeness is assessed using cluster analysis, the which aim is to separate the input data set *X* = {*x*_1_, *x*_2_, …, *x_n_*} (see Table 2, Table 3 and Table 4) into *k* disjoint clusters, where *k* is a predetermined number of clusters (*k* = 8 is assumed based on [59,60,61]). Each cluster is an object group that is considered more similar than objects from other clusters. Taking into account the results of [64], the *k*-means cluster analysis method was applied, based on minimizing the squared distances sum between cluster objects and their centroids (the *j*-th cluster centre) *C* = {*μ*_1_, *μ*_2_, …, *μ_n_*}, where $\mu_{j}\in R^{d}$.

For each *x_i_* value of helicopter thermogas-dynamic parameters TE *n_TC_*, $T_{G}^{*}$ and *n_FT_*, the distance to all centroids is calculated and the object is assigned to the cluster with the nearest centroid according to the expression:

(36) $$r_{ij}=\left\{\begin{array}{c} 1,\;\mathrm{if}\;j=\arg\min_{l}{\left\|x_{i}-\mu_{l}\right\|}^{2},\\ 0,\;\mathrm{otherwise} \end{array}\right.$$

where $j=\arg\min_{l}{\left\|x_{i}-\mu_{l}\right\|}^{2}$ means that object *x_i_* belongs to the *j*-th cluster, with $r_{ij}\in \left\{0,1\right\}$.

The *k*-means method minimizes the squared distance sum between features and their corresponding centroids. The objective function is presented as:

(37) $$J=\sum_{j=1}^{k}\sum_{i=1}^{N}r_{ij}\cdot {\left\|x_{i}-\mu_{j}\right\|}^{2}.$$

For each *j*-th cluster, the centroid *μ_j_* is updated as all objects assigned to that cluster average as:

(38) $$\mu_{j}=\frac{\sum_{i=1}^{N}r_{ij}\cdot x_{i}}{\sum_{i=1}^{N}r_{ij}}.$$

Calculations according to (36) and (38) are repeated until the object assignments to clusters stop changing (until convergence).

**Figure 8:**
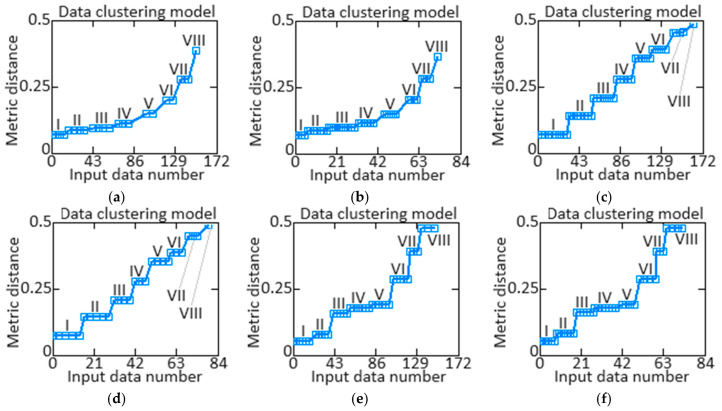
Cluster analysis results: (**a**) training sample of the parameter *n_TC_*, (**b**) test sample of the parameter *n_TC_*, (**c**) training sample of the parameter $T_{G}^{*}$, (**d**) test sample of the parameter $T_{G}^{*}$, (**e**) training sample of the parameter *n_FT_*, (**f**) test sample of the *n_FT_* parameter (author’s research).

A random selection procedure was used to select training and test samples in a 2:1 ratio (67 and 33%, respectively—172 and 84 elements) based on the helicopter TE (see Table 2, Table 3 and Table 4) thermogas-dynamic parameter training samples. The cluster data analysis results from the helicopter TE thermogas-dynamic parameter (Table 2, Table 3 and Table 4) training samples revealed 8 classes (classes I…VIII), that is, eight groups are present in them, which indicates the composition similarity of both training and test samples (Figure 8).

The results obtained made it possible to determine the helicopter TE thermogas-dynamic parameters’ optimal sample size: the training sample is 256 elements (100%), the control sample is 172 elements (67% of the training sample), and the test sample is 84 elements (33% of the training sample).

### 3.2. The Developed Neural Network Training Results

**Figure 9:**
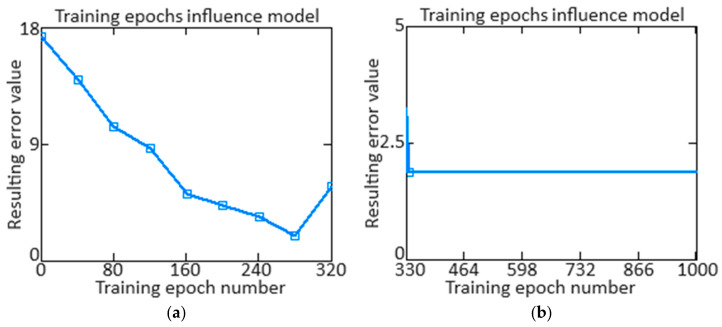
The influence diagram for the number of epochs passed on the resulting error (author’s research). (**a**) Training for the 320 epochs (**b**) Training from 320 to 1000 epochs.

At the developed neural network (see Figure 6) training in the 1st stage, the epochs passed number (Figure 9, Table 5) which influence the final standard deviation that is the training (loss function) quality assessing criterion and is defined as [59,60,61]:

(39) $$E_{epoch}=\frac{1}{N}\cdot \sum_{i=1}^{N}\left(\frac{1}{2}\cdot \sum_{k=1}^{n}{\left(y_{k}-{\hat{y}}_{k}\right)}^{2}\right).$$

The results obtained indicate that 280 training epochs are sufficient to achieve the minimum value of *E_epoch_* = 2.005. (Figure 9a). It is worth noting that after 280 epochs, the neural network training error increases. The neural network training convergence demonstrated it was trained for 1000 epochs (Figure 9b). It can be seen that almost immediately after 320 training epochs, the loss function decreases to its minimum value *E_epoch_* = 2.005 and remains stable over 1000 training epochs. The slight increase in training error after epoch 280 is due to the overfitting phenomenon, where the model begins to overfit the training data rather than generalize to the new data. However, after epoch 330, the error is reduced again due to hyperparameter adjustments using regularization techniques that stabilize training.

The situation described above, where a slight increase in the training error is observed after the *i*-th epoch due to model overtraining, is discussed in detail in [65,66]. These sources provide various methods for reaching this state, including adjusting hyperparameters and using regularization methods. These methods aim to stabilize the training process and improve the model’s generalisation ability. It is important to note that the temporary increase in training error is not critical and can be effectively managed using appropriate techniques, allowing the model to continue training and achieve optimal results.

**Figure 10:**
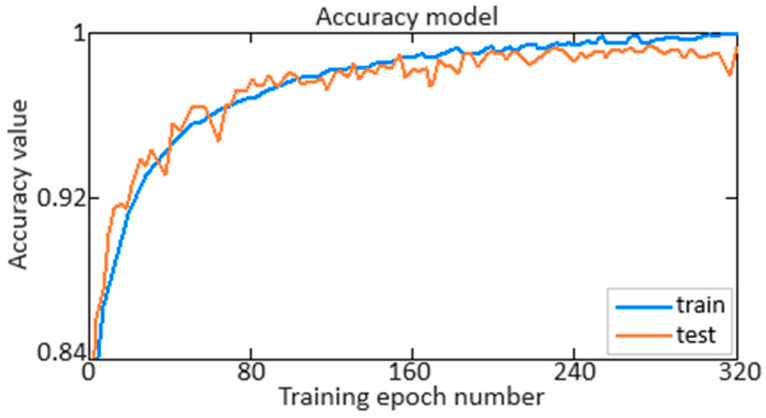
Accuracy metric diagram (author’s research).

**Figure 11:**
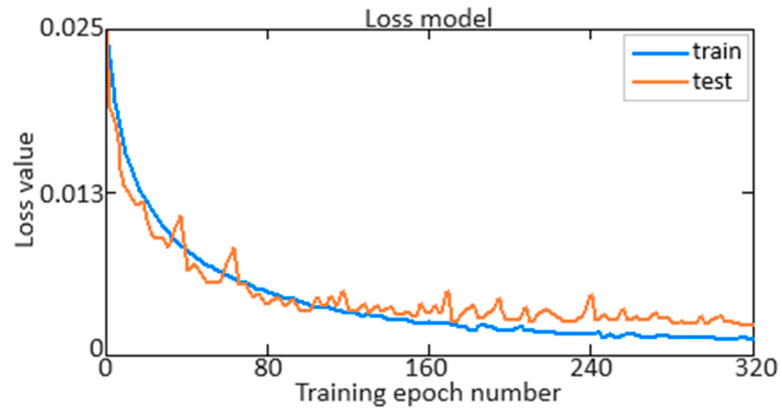
Loss function diagram (author’s research).

At the next stage of training of the developed neural network (see Figure 6), its performance, accuracy (Figure 10) and loss (Figure 11) are determined for 280 training epochs. Accuracy provides information about how well the model classifies the data, while loss reflects how well the model minimises the difference between predicted and actual values. 

As can be seen from Figure 10, accuracy reaches a value of 0.995 (almost 100%). Moreover, as can be seen from Figure 11, the loss function does not exceed 0.025 (2.5%) at the beginning of training and decreases to 0.005 (0.5%) at 280 training epochs. This indicates the high efficiency and accuracy of the developed neural network in solving the problem. Achieving an accuracy value of 0.995 (almost 100%) indicates that the model successfully classifies the data with high accuracy. In parallel, the observed decrease in the loss function from 0.025 (2.5%) to 0.005 (0.5%) indicates that the model successfully reduces the difference between the actual and predicted values, which is a key indicator of the effectiveness of neural network training. These results highlight the high-quality performance of the model and its ability to make accurate and reliable predictions.

It is worth noting that the work compares the accuracy and loss definition. As mentioned above, accuracy reaches a value of 0.995, and the loss function decreases from 0.025 (2.5%) to 0.005 (0.5%) after 280 training epochs when applying the Smooth ReLU activation function. At the same time, when using the ReLU activation function, accuracy reaches a 0.995 value when the loss function is reduced from 0.025 (2.5%) to 0.005 (0.5%) and is achieved with 490 training epochs, which is almost several times further compared to using Smooth ReLU. Moreover, with 280 training epochs, when using the ReLU activation function, accuracy only reaches a 0.972 value, and the loss function decreases from 0.025 (2.5%) to 0.018 (1.8%).

### 3.3. Helicopter Turboshaft Engines Thermogas-Dynamic Parameter Sensors Signal Neural Network Integration Results

**Figure 12:**
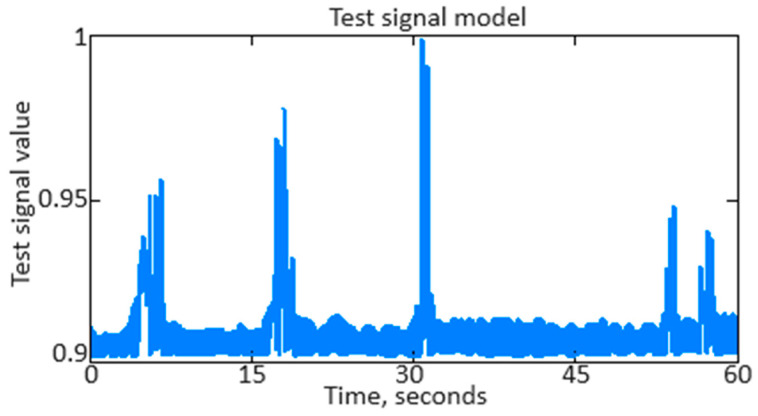
Initial diagram of the *n_TC_* gas–generator rotor rpm signal (author’s research).

To conduct a computational experiment based on helicopter TE thermogas-dynamic parameter (see Table 2, Table 3 and Table 4) training samples, as an example, the *n_TC_* parameter original signal, contaminated with noise, that was received from the corresponding sensor (Figure 12) was restored.

**Figure 13:**
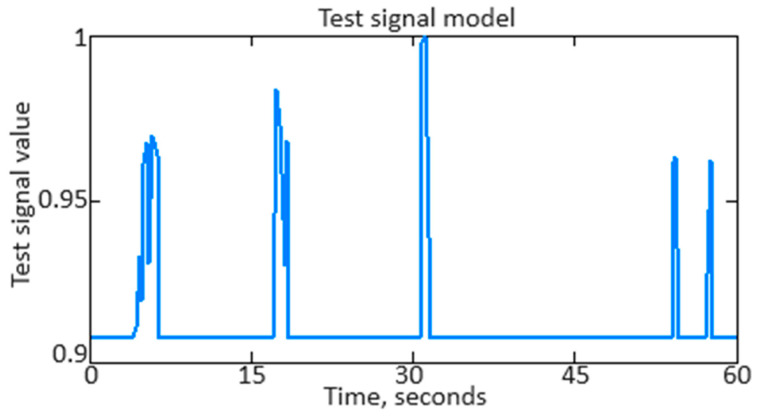
Resulting diagram of the *n_TC_* gas–generator rotor rpm signal (author’s research).

As can be seen from Figure 12, the original nTC parameter signal contains various distortions, interference, and noise, which can affect its accuracy in analysis and interpretation. Figure 13 shows the *n_TC_* parameter filtered signal after applying the developed neural network (see Figure 6). 

As shown in Figure 13, the *n_TC_* parameter signal filtering effectively removed noise and distortion while the signal’s main characteristics were preserved. The filtered *n_TC_* signal appears cleaner and smoother, making it more suitable for further analysis and use.

**Figure 14:**
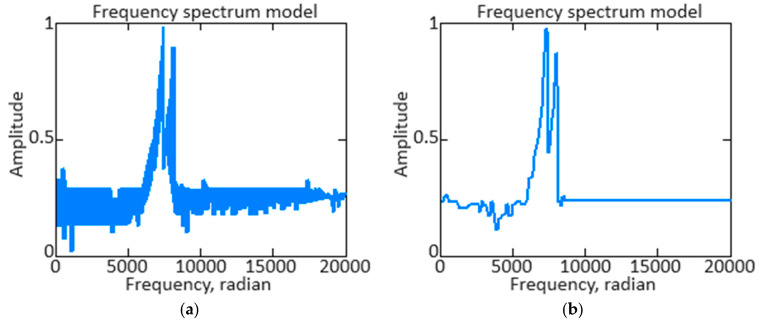
The *n_TC_* gas–generator rotor rpm signal spectrum diagram: (**a**) Original signal (**b**) Filtered signal (author’s research).

To analyze the signal frequency composition, the *n_TC_* parameter is used to isolate or suppress certain frequency components using the direct Fourier transform $F\left(\omega \right)=\int_{-\infty }^{\infty }f\left(t\right)\cdot e^{-j\cdot \omega \cdot t}dt$ the transition from the time domain to the frequency domain has been completed. This allows you to determine which frequencies are present in the signal and at what amplitude, which in turn helps determine which frequencies should be retained or excluded to achieve the desired filtering result. Moving into the frequency domain allows you to understand the signal structure better and make informed decisions about the necessary filtering actions, such as noise reduction, extraction of frequency components of interest, or interference suppression. Figure 14a shows the *n_TC_* parameter’s original signal spectrum, and Figure 14b shows the *n_TC_* parameter’s filtered signal spectrum.

The *n_TC_* parameter is the original and filtered signals spectrum resulting diagram, allowing us to compare their frequency characteristics visually. Analyzing the differences between the spectra allows you to determine the filtration effectiveness and evaluate how successfully your aims were achieved. If the filtered signal spectrum shows a significant reduction in amplitude at frequencies that need to be suppressed and retention or enhanced that are important to the signal, this indicates the designed neural network is (Figure 6) functioning well.

**Figure 15:**
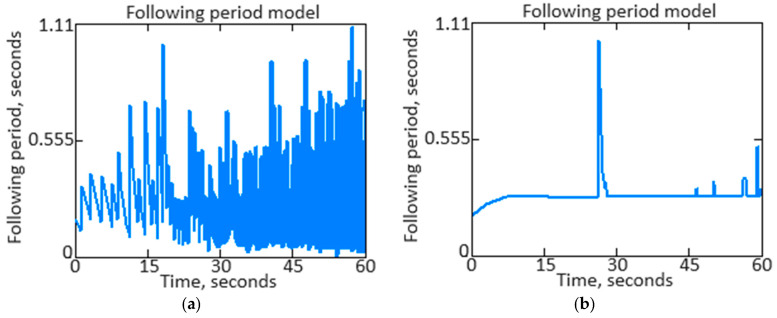
The *n_TC_* gas–generator rotor rpm signal repetition period diagram: (**a**) Original signal (**b**) Filtered signal (author’s research).

Further in the work, the parameter *n_TC_* signal repetition period is determined as $T=\frac{1}{f}$, where $f=\frac{2\pi }{\omega }$ is the frequency. This allows you to understand the *n_TC_* parameter signal time’s main characteristics, such as its frequency and frequency spectrum. Knowing the signal period helps analyze its dynamics and detect regular patterns. Figure 15a shows the parameter *n_TC_* initial signal repetition period, and Figure 15b shows the *n_TC_* parameter filtered signal repetition period.

At the computational experiment next stage, a transition was made from the parameter signal *n_TC_*, presented in the time dependence *f*(*t*) form (see Figure 12), to the signal-to-noise ratio (*SNR*) according to the expression:

(40) $$SNR=\frac{\int_{0}^{T}{\left(f\left(t\right)\right)}^{2}}{\int_{0}^{T}{\left(n\left(t\right)\right)}^{2}},$$

where *n*(*t*) is the noise signal (in this work it is taken as a random variable).

**Figure 16:**
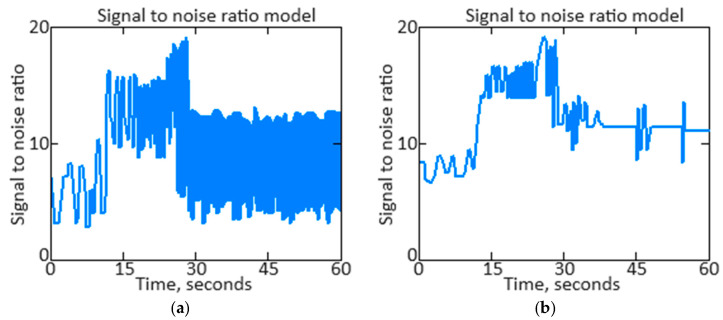
The *n_TC_* gas–generator rotor rpm signal signal-to-noise ratio diagram: (**a**) Original signal (**b**) Filtered signal (author’s research).

*SNR* provides a clear numerical value that shows how much the signal stands out from the background noise. A high *SNR* indicates that the signal dominates the noise, which means better data transmission and processing. A low *SNR* indicates that noise is having a significant impact, which can result in distortion or loss of important information. Figure 16a shows the *SNR* based on the original signal of the *n_TC_* parameter, and Figure 16b shows the *SNR* based on the filtered signal of the *n_TC_* parameter.

**Figure 17:**
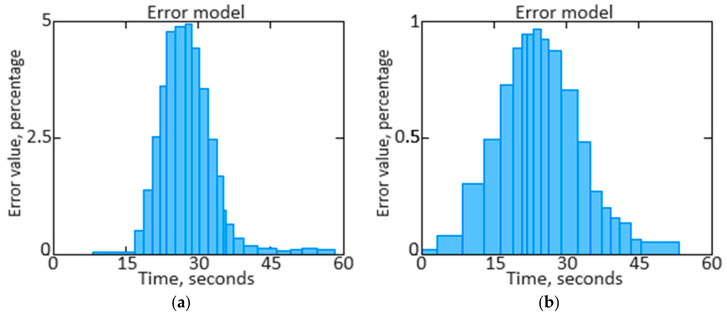
Signal histogram for the *n_TC_* gas–generator rotor rpm estimates: (**a**) Original signal (**b**) Filtered signal (author’s research).

**Figure 18:**
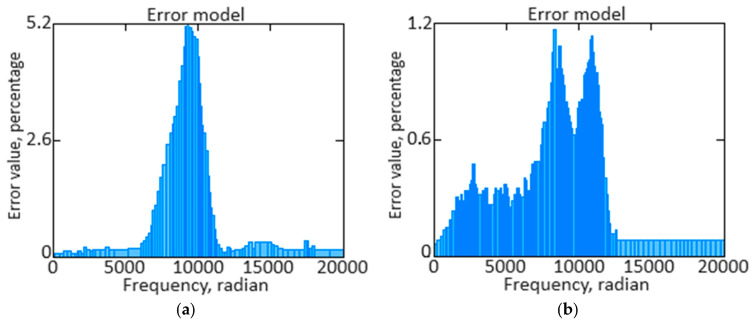
The spectrum histogram for the *n_TC_* gas–generator rotor rpm signal estimates: (**a**) Original signal (**b**) Filtered signal (author’s research).

**Figure 19:**
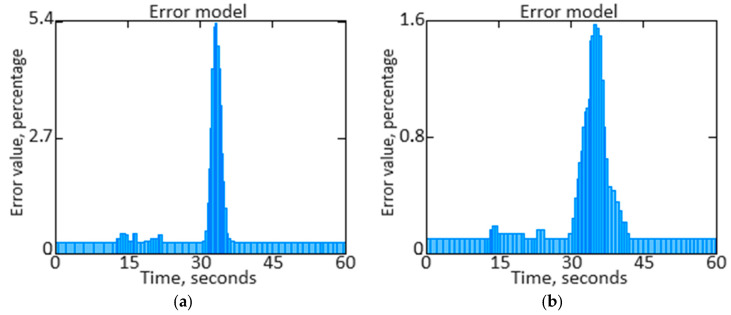
The sequence histogram for the gas–generator rotor rpm signal *n_TC_* estimates: (**a**) Original signal (**b**) Filtered signal (author’s research).

**Figure 20:**
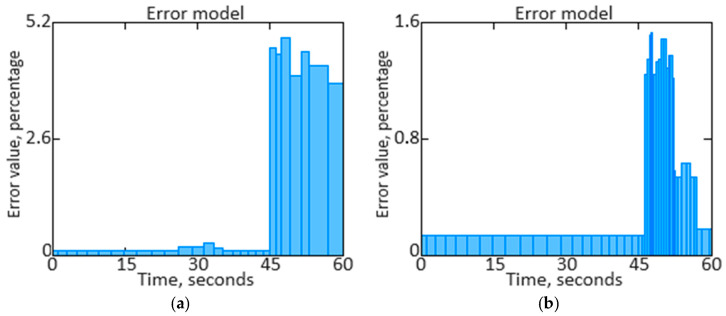
The *n_TC_* gas–generator rotor rpm signal signal/noise estimates histogram: (**a**) Original signal (**b**) Filtered signal (author’s research).

We have performed a comparison of filtered and unfiltered results histograms along with the *n_TC_* parameter signal filtered estimates dynamics qualitative analysis (Figure 17, Figure 18, Figure 19 and Figure 20). 

The obtained data analysis (see Figure 17, Figure 18, Figure 19 and Figure 20) allows us to state that combining signals using the filtering method leads to narrowing the unfiltered estimate histogram, which, in turn, helps to increase accuracy. The presented histogram research shows that the histogram maximum narrowing, and therefore accuracy improvement, is achieved for the pulse repetition period.

## 4. Discussion

### 4.1. Noise Variance Estimation

The *n_TC_* parameter signal (see Figure 12 and Figure 13) noise dispersion *f*(*t*) estimation is an important aspect of time series analysis and signal processing since dispersion characterizes the noise values spread degree around its average value. In the signal *f*(*t*) analyzing process, which can be represented as the useful signal and noise sum, isolating and estimating the noise variance makes it possible for the filtering methods to signal and the effectiveness quality evaluated. A statistical method is used to calculate noise variance: first, the average noise value is determined, and then all noise values’ standard deviation from this average is calculated. Noise dispersion is defined as:

(41) $$\sigma^{2}=\frac{1}{N}\cdot \sum_{i=1}^{N}{\left(n\left(t\right)-\bar{n}\right)}^{2},$$

where *n*(*t*) are the noise values at time *t*, $\bar{n}$ is the average noise value, and *N* is the element number in the training set.

**Figure 21:**
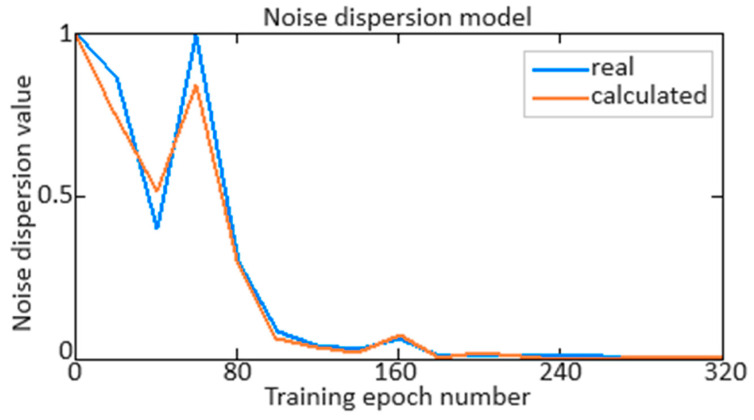
Noise dispersion diagram of the *n_TC_* gas–generator rotor rpm signal (author’s research).

Figure 21 shows a diagram of the noise dispersion resulting estimate depending on the number of the elements in the training set, from which it can be seen that if there are 156 elements in the training set (58% of the total volume), the noise dispersion value becomes almost equal to zero. This indicates that increasing the training set size significantly improves the model’s accuracy in estimating noise, and once a certain amount of data are reached, the model almost eliminates the uncertainties associated with noise. Thus, we can conclude that to achieve minimum noise variance, it is necessary to use at least 156 elements in the training set, which ensures that all possible signal variations have adequate representation and allows the model to effectively take them into account.

### 4.2. Comparative Analysis of Neural Network Signal Integration Based on the Filtering Method with Traditional Filters

When performing the neural network signal integration comparative analysis using the filtering method and traditional filters, the following metrics are used:

1. Mean square error characterizes the differences in squares arithmetic mean between the observed and predicted values defined as:

(42) $$MSE=\frac{1}{N}\cdot \sum_{i=1}^{N}{\left(f_{i}\left(t\right)-{\hat{f}}_{i}\left(t\right)\right)}^{2}.$$

2. The average absolute error characterizes the estimated values’ average absolute deviation from the true signal values and is defined as:

(43) $$MAE=\frac{1}{N}\cdot \sum_{i=1}^{N}\left|f_{i}\left(t\right)-{\hat{f}}_{i}\left(t\right)\right|.$$

3. The determination coefficient is a measure showing the variation proportion in the dependent variable explained by the independent variables in the model and is defined as:

(44) $$R^{2}=1-\frac{\frac{1}{N}\cdot \sum_{i=1}^{N}{\left(f_{i}\left(t\right)-{\hat{f}}_{i}\left(t\right)\right)}^{2}}{\frac{1}{N}\cdot \sum_{i=1}^{N}{\left(f_{i}\left(t\right)-\bar{f}\right)}^{2}}.$$

4. Peak signal to noise is used to the reconstructed signal quality measure concerning the maximum signal and is defined as:

(45) $$PNSR=10\cdot \log_{10}\left(\frac{{MAX}^{2}}{MSE}\right),$$

where *MAX* is the maximum possible signal value.

5. Signal-to-noise ratio measures the ratio between signal power and noise power and is defined as:

(46) $$SNR=10\cdot \log_{10}\left(\frac{\sum_{i=1}^{N}{\left(f_{i}\left(t\right)\right)}^{2}}{\sum_{i=1}^{N}{\left(f_{i}\left(t\right)-{\hat{f}}_{i}\left(t\right)\right)}^{2}}\right).$$

6. Correlation measures the linear relations between the true and filtered values and is defined as:

(47) $$r=\frac{\frac{1}{N}\cdot \sum_{i=1}^{N}\left(f_{i}\left(t\right)-\bar{f}\right)\cdot \left({\hat{f}}_{i}\left(t\right)-\bar{\hat{f}}\right)}{\sqrt{\sum_{i=1}^{N}{\left(f_{i}\left(t\right)-\bar{f}\right)}^{2}\cdot \sum_{i=1}^{N}{\left(f_{i}\left(t\right)-\bar{\hat{f}}\right)}^{2}}},$$

where $\bar{f}$ and $\bar{\hat{f}}$ are the average values of the true and filtered signals, respectively.

7. The root mean square error is the square root of the *MSE* gives an idea of magnitude errors and is defined as:

(48) $$RMSE=\sqrt{\frac{1}{N}\cdot \sum_{i=1}^{N}{\left(f_{i}\left(t\right)-{\hat{f}}_{i}\left(t\right)\right)}^{2}}.$$

8. Average absolute percent error measures the average absolute error as the percentage of the true value and is defined as:

(49) $$MAPE=\frac{100\%}{N}\cdot \sum_{i=1}^{N}\left|\frac{f_{i}\left(t\right)-{\hat{f}}_{i}\left(t\right)}{f_{i}\left(t\right)}\right|.$$

9. Average relative error estimates the predicted values average relative error relative to the true values and is defined as:

(50) $$MRE=\frac{1}{N}\cdot \sum_{i=1}^{N}\left|\frac{f_{i}\left(t\right)-{\hat{f}}_{i}\left(t\right)}{f_{i}\left(t\right)}\right|.$$

10. The goodness-of-fit index measures the agreement between the true and predicted values, taking into account both precision and deviation and is defined as:

(51) $$CCC=\frac{2\cdot r\cdot \sigma_{f}\cdot \sigma_{\hat{f}}}{\sigma_{f}^{2}+\sigma_{\hat{f}}^{2}+{\left(\bar{f}-\bar{\hat{f}}\right)}^{2}},$$

where *σ_f_* and $\sigma_{\hat{f}}$ are the true and predicted values standard deviations, respectively.

11. Normalized mean squared error normalizes the true values variance *MSE* concerning, allowing comparison of models at different data scales and is defined as:

(52) $$NMSE=\frac{\frac{1}{N}\cdot \sum_{i=1}^{N}{\left(f_{i}\left(t\right)-{\hat{f}}_{i}\left(t\right)\right)}^{2}}{\frac{1}{N}\cdot \sum_{i=1}^{N}{\left(f_{i}\left(t\right)-\bar{f}\right)}^{2}}.$$

12. The signal reconstruction quality function evaluates the signal reconstruction quality, taking into account the minimum values between the true and predicted values and is defined as:

(53) $$SQR=\frac{\frac{1}{N}\cdot \sum_{i=1}^{N}\min\left(f_{i}\left(t\right),{\hat{f}}_{i}\left(t\right)\right)}{\frac{1}{N}\cdot \sum_{i=1}^{N}f_{i}\left(t\right)}.$$

Table 6 shows the neural network signal integration based on the filtering method with recursive [67], median [68] and median-recursive [69] filters comparative analysis results according to metrics (42)–(53).

The results obtained (see Table 6) confirm that neural network signal integration based on the filtering method is the best in all metrics, demonstrating minimal errors and maximum correspondence to the true signal. Meanwhile, applying the median filter is the worst, showing maximum errors and minimum compliance. Table 7 shows the improvement in the neural network quality metrics signal integration results based on the filtering method with traditional filters.

### 4.3. Results of a Trained Neural Network with Traditional Filtering Methods Comparison

To compare the trained neural network model (Figure 6) for each layer data with the traditional filtering method (for example, using a median-recursive filter), each step results, followed by checking each hidden layer design’s actual effectiveness. The following algorithm is used in the work.

The training samples’ data (Table 2, Table 3 and Table 4) are divided into signals and noise to apply traditional filtering methods, followed by the results’ analysis at each step. In this case, *S_clean_* = {*x*_1_, *x*_3_, *x*_5_} is a pure signals’ set (*x*_1_ is the gas–generator rotor rpm *n_TC_* signal; *x*_3_ is the gas temperature in the compressor turbine $T_{G}^{*}$ signal front; and *x*_5_ is the free turbine rotor speed *n_FT_* signal), and *N_noice_* = {*x*_2_, *x*_4_, *x*_6_} is the noise’s set (*x*_2_ is the gas–generator rotor rpm *n_TC_* noice; *x*_4_ is the gas temperature in the compressor turbine $T_{G}^{*}$ noise front; and *x*_6_ is the free turbine rotor speed *n_FT_* noise). As a result, two sets are obtained: one containing pure signals and the other containing their corresponding noise.

Next, the traditional filtering method performs each step on the prepared data. In this case, signals are sequentially filtered from interference, dynamic changes are compensated, and sequential filtering is carried out to reduce noise and highlight useful signals.

After this, the prepared data are passed through a trained neural network, followed by saving each hidden layer output: the 1st hidden layer outputs (summing signals with noise) are the *h*_1_, *h*_2_, *h*_3_; the 2nd hidden layer outputs (dynamic compensation) are the *z*_1_, *z*_2_, and *z*_3_; the 3rd hidden layer outputs (filtering first stage) are the *f*_1_, *f*_2_, and *f*_3_; the 4th hidden layer outputs (filtering second stage) are the *g*_1_, *g*_2_, and *g*_3_; and the output layer is the *y*.

The following is a direct comparison:

1. The summing signals with noise results by the traditional method are compared with the neural network’s 1st hidden layer results.
2. The dynamic compensation results by the traditional method are compared with the neural network’s 2nd hidden layer results.
3. The filtering 1st stage results by the traditional method are compared with the neural network’s 3rd hidden layer results.
4. The filtering 2nd stage results by the traditional method are compared with the neural network’s 4th hidden layer results.

In a computational experiment, clear signals and noise interference were identified from the training samples data (Table 2, Table 3 and Table 4), which are shown in Table 8, Table 9 and Table 10.

Table 11 shows the data processing results (Table 8, Table 9 and Table 10) using a neural network. Table 12 uses traditional filtering methods according to stages.

The output parameter value obtained in the neural network for each layer is significantly higher than the values obtained using traditional filtering methods, consisting of summing signals with their noise, dynamic compensation, and the first and second filtering stages (see Table 13). This is because neural networks can train and adapt to data complex and nonlinear relations, allowing them to compensate for interference and improve signal quality more effectively. Having the Smooth ReLU activation function allows the neural network to ignore negative values that may represent noise and emphasize positive values, thereby improving the output signal quality. However, higher output values indicate a cleaner and more amplified signal, which is important for improving the overall system accuracy and reliability.

### 4.4. The I and II Type Errors Calculation

A type I error occurs when the null hypothesis *H*_0_ is rejected even though it is true, and is defined at a given significance level as:

(54) $$\alpha =P\left(Reject\;H_{0}|H_{0}\;is\;true\right).$$

A type II error occurs when the null hypothesis *H*_0_ is not rejected even though the alternative hypothesis *H*_1_ is true, and is defined as:

(55) $$\beta =P\left({Don}^{\prime }t\;reject\;H_{0}|H_{1}\;is\;true\right).$$

As mentioned above, the paper set the significance level to 0.01, which means the type I error probability (erroneously rejecting a true null hypothesis) is 1%; that is, if a statistical test shows a significant result, there is only a 1% chance that this result is due to chance and caused by noise or random variations in the data. The significance indicates a given level of high requirements for the reliability of the results, which is especially important for ensuring the helicopter TE thermogas-dynamic parameters sensor readings’ accuracy and reliability.

For the given task, the null hypothesis is “The developed neural network (see Figure 6) does not improve the helicopter TE thermogas-dynamic parameters from sensor signals integration accuracy in comparison with traditional filters (median-recursive, recursive and median filters)”, and the alternative hypothesis is “The developed neural network (see Figure 6) significantly improves the helicopter TE thermogas-dynamic parameter sensor signals integrating accuracy compared to traditional filters (median-recursive, recursive and median filters)”.

Table 8 shows the 1st and 2nd types’ errors calculating results for neural network integration of signals based on the filtering method with recursive [67], median [68] and median-recursive [69] filters according to metrics (42)–(53).

The results (see Table 14) showed that the neural network signal integration based on the filtering method used made it possible to reduce the 1st and 2nd types’ errors by 2.11 times compared with the use of a median-recursive filter, by 2.89 times compared with the recursive filter use, and by 4.18 times compared with the recursive filter use. using a median filter.

The neural network implementation approach in real helicopter operating conditions faces challenges and advantages number. The main challenges include the need for significant computing resources to train and operate the neural network, difficulties adapting to rapidly changing operating conditions, and interference of various types. In addition, careful model tuning and validation of large amounts of data are required to avoid overfitting and ensure that the system operates stably. However, this approach’s advantages are significant: it provides helicopter TE parameters signal filtering with higher accuracy and reliability through adaptive noise suppression and integration of dynamic compensation methods. A neural network trained using a backpropagation algorithm with an adaptive training rate allows you to balance between model accuracy and generalization ability, preventing overfitting. As a result, this method improves the signal filtering efficiency compared to traditional methods and reduces errors of the first and second types several times, significantly increasing the helicopter TE performance and reliability control in real operating conditions.

Thus, the research did not focus on creating a new helicopter or engine. At the same time, it is focused on the sensors data analysis from a particular class of existing helicopter TE thermogas-dynamic parameters (the TV3-117 engine was used in the work) and its parameters (Table 2, Table 3 and Table 4 [40,41,42,59,60,61]), obtained in the Mi-8MTV helicopters flight operation. This aim is achieved by collecting data from sensors during engine operation, data analysis obtained to identify deviations in engine operation, comparison of deviations with benchmarks for a specific engine type, and determining the resulting deviations’ causes.

The prospect for further research is the recommendations for eliminating the identified deviations of helicopter TE parameters from the reference values.

## 5. Conclusions

The article develops a helicopter turboshaft engine’s thermogas-dynamic parameter signals neural network integration method, which allows data to be effectively corrected from sensors in real time, ensuring high accuracy and reading reliability:

1. The helicopter turboshaft engines’ thermogas-dynamic parameter signals neural network integration method relevance is substantiated since this method provides effective noise filtering, which makes it possible to increase the engine condition monitoring accuracy.
2. An integrating signals scheme from helicopter turboshaft engine thermogas-dynamic parameter sensors has been developed using a filtering method, which achieves almost 100% (0.995 or 99.5%) accuracy and reduces the loss function to 0.005 (0.5%) with 280 training epochs.
3. Based on the backpropagation algorithm, a neural network training method has been developed for the helicopter turboshaft engine parameters integrating control loops, which combines increasing accuracy on the validation sample and controlling overtraining into a single criterion. This method minimizes the loss function and considers the error dynamics on the validation set, preserving the model’s ability to generalize. The adaptive training rate helps quickly adapt to data changes and improves performance. In this case, to achieve the loss function minimum value of 2.005, 280 training epochs are enough, after which the error begins to increase; however, the loss function stabilizes immediately after 320 epochs and remains stable for 1000 epochs.
4. It is proposed that a modified Smooth ReLU activation function be used, in which accuracy reaches 0.995, and the loss function decreases from 0.025 to 0.005 in 280 epochs, while with ReLU it takes 490 epochs to achieve the same accuracy and loss, and in 280 epochs the accuracy reaches only 0.972. Furthermore, losses are reduced to 0.018.
5. It is mathematically substantiated that the neural network integration closed loops used for regulating the helicopter turboshaft engine parameters using the filtering method compared with traditional filters (median-recursive, recursive, median filter) improves efficiency by 1.020…5.101 times compared to the median-recursive filter, 1.031…9.658 times compared to the recursive filter, and 1.082…20.325 times compared to the median filter.
6. It is mathematically substantiated that the neural network signal integration use based on the filtering method made it possible for the first and second types to reduce errors by 2.11 times compared with the median-recursive filter use, by 2.89 times compared with the recursive filter use, and by 4.18 times compared with the median filter use.

## Figures and Tables

**Table 1:** Analytical expressions for the developed neural network hidden layers parameters calculating (author’s research).

Neural Network Layer	Parameter	Analytical Expression
4th hidden layer	Neuron weight error	$\delta_{g_{i}}=\delta_{out}\cdot w_{ij}^{\left(4\right)},$ where $w_{ij}^{\left(4\right)}$ mean the 4th hidden layer of neurons’ weights (see expression (13)).
	Gradient error	$\delta_{g_{i}}=\delta_{g_{i}}\cdot \frac{\partial \left(Smooth\;ReLU\right)}{\partial x},$ where $\frac{\partial \left(Smooth\;ReLU\right)}{\partial x}$ is defined according to (27).
	Gradients by weights	$\frac{\partial E}{\partial w_{ij}^{\left(4\right)}}=\delta_{g_{i}}\cdot f_{i}$
3rd hidden layer	Neuron weight error	$\delta_{f_{i}}=\sum \delta_{f_{i}}\cdot w_{ij}^{\left(3\right)},$ where $w_{ij}^{\left(3\right)}$ mean the 3rd hidden layer neurons weights (see expression (12)).
	Gradient error	$\delta_{f_{i}}=\delta_{f_{i}}\cdot \frac{\partial \left(Smooth\;ReLU\right)}{\partial x},$ where $\frac{\partial \left(Smooth\;ReLU\right)}{\partial x}$ is defined according to (27).
	Gradients by weights	$\frac{\partial E}{\partial w_{ij}^{\left(3\right)}}=\delta_{f_{i}}\cdot z_{i}$
2nd hidden layer	Neuron weight error	$\delta_{z_{i}}=\sum \delta_{z_{i}}\cdot w_{ij}^{\left(2\right)},$ where $w_{ij}^{\left(2\right)}$ mean the 3rd hidden layer of neurons’ weights (see expression (11)).
	Gradient error	$\delta_{z_{i}}=\delta_{z_{i}}\cdot \frac{\partial \left(Smooth\;ReLU\right)}{\partial x},$ where $\frac{\partial \left(Smooth\;ReLU\right)}{\partial x}$ is defined according to (27).
	Gradients by weights	$\frac{\partial E}{\partial w_{ij}^{\left(2\right)}}=\delta_{z_{i}}\cdot h_{i}.$

**Table 2:** The training sample fragment for *n_TC_* gas–generator rotor rpm (author’s research).

Number	1	2	…	37	…	84	…	115	…	172	…	202	…	256
**Value**	0.943	0.982	…	0.948	…	0.957	…	0.962	…	0.974	…	0.935	…	0.981

**Table 3:** The training sample fragment for $T_{G}^{*}$ gas temperature in the compressor turbine front (author’s research).

Number	1	2	…	29	…	73	…	109	…	164	…	200	…	256
**Value**	0.932	0.964	…	0.975	…	0.926	…	0.918	…	0.905	…	0.902	…	0.953

**Table 4:** The training sample fragment for *n_FT_* free turbine rotor speed (author’s research).

Number	1	2	…	32	…	80	…	105	…	181	…	207	…	256
**Value**	0.929	0.933	…	0.909	…	0.932	…	0.941	…	0.955	…	0.926	…	0.973

**Table 5:** Determining the influence results for the number of epochs passed on the resulting error (author’s research).

Number	1	2	3	4	5	6	7	8	9
**Epoch**	0	40	80	120	160	200	240	280	320
***E_epoch_***	17.352	14.018	10.342	8.665	5.229	4.315	3.399	2.005	3.767

**Table 6:** The neural network signal integration based on the filtering method with traditional filters comparative analysis results (author’s research).

Metrics	Neural Network Integration	Median Recursive Filter	Recursive Filter	Median Filter
*MSE*	0.000992	0.00255	0.00912	0.0116
*MAE*	0.0079	0.0403	0.0763	0.1622
*R* ^2^	0.9495	0.7358	0.6892	0.5171
*PNSR*	40.02 dB	25.91 dB	20.38 dB	13.80 dB
*SNR*	39.35 dB	25.25 dB	19.72 dB	13.13 dB
*r*	0.9761	0.6519	0.4219	0.2740
*RMSE*	0.0315	0.0505	0.0955	0.1077
*MAPE*	0.8615%	1.365%	4.250%	17.50%
*MRE*	0.00861	0.0436	0.0825	0.1750
*CCC*	0.9756	0.6009	0.4998	0.1097
*NMSE*	0.505	1.299	2.641	4.121
*SQR*	0.9960	0.9766	0.9656	0.9207

**Table 7:** The neural network signal integration improvement degree calculating results based on the filtering method with traditional filters (author’s research).

Metrics	The Improvement Compared to the Median Recursive Filter	The Improvement Compared to the Recursive Filter	The Improvement Compared to the Median Filter
*MSE*	2.571	9.194	11.694
*MAE*	5.101	9.658	20.532
*R* ^2^	1.290	1.378	1.836
*PNSR*	1.545	1.964	2.900
*SNR*	1.558	1.995	2.997
*r*	1.497	2.314	3.562
*RMSE*	1.603	3.032	3.419
*MAPE*	1.584	4.933	20.313
*MRE*	5.064	9.582	20.325
*CCC*	1.624	1.952	8.893
*NMSE*	2.572	5.230	8.160
*SQR*	1.020	1.031	1.082

**Table 8:** The training sample fragment for *n_TC_* gas–generator rotor rpm with the separation of clean signal and noise interference (author’s research).

Number	1	2	…	37	…	84	…	115	…	172	…	202	…	256
***S_clean_***	0.922	0.962	…	0.923	…	0.935	…	0.942	…	0.944	…	0.912	…	0.956
***N_noice_***	0.021	0.020	…	0.025	…	0.022	…	0.020	…	0.030	…	0.023	…	0.025

**Table 9:** The training sample fragment for $T_{G}^{*}$ gas temperature in the compressor turbine front with the separation of clean signal and noise interference (author’s research).

Number	1	2	…	29	…	73	…	109	…	164	…	200	…	256
***S_clean_***	0.903	0.933	…	0.955	…	0.895	…	0.898	…	0.875	…	0.880	…	0.922
***N_noice_***	0.029	0.031	…	0.020	…	0.031	…	0.020	…	0.030	…	0.022	…	0.031

**Table 10:** The training sample fragment for *n_FT_*-free turbine rotor speed with the separation of clean signal and noise interference (author’s research).

Number	1	2	…	32	…	80	…	105	…	181	…	207	…	256
***S_clean_***	0.907	0.913	…	0.888	…	0.911	…	0.921	…	0.936	…	0.903	…	0.952
***N_noice_***	0.022	0.020	…	0.021	…	0.021	…	0.020	…	0.019	…	0.023	…	0.021

**Table 11:** Data processing results from training samples using a neural network (author’s research).

Stage Number	Stage Name	Results
1	1st hidden layer	Parameters *h*_1_, *h*_2_, *h*_3_ are calculated according to (10). The final values are *h*_1_ = 0.898, *h*_2_ = 0.875, *h*_3_ = 0.880.
2	2nd hidden layer	The accepted weight and bias matrices are: $W=\left(\begin{array}{ccc} 0.5 & 0.3 & 0.2\\ 0.4 & 0.6 & 0.1\\ 0.3 & 0.3 & 0.4 \end{array}\right)$, $b=\left(\begin{array}{c} 0.1\\ 0.2\\ 0.1 \end{array}\right)$. Using the Smooth ReLU activation function, the parameters *z*_1_, *z*_2_, *z*_3_ are calculated according to (11). The final values are *z*_1_ = 0.988, *z*_2_ = 1.172, *z*_3_ = 0.984.
3	3rd hidden layer	By applying the Smooth ReLU activation function to the linear combinations *z_i_*, the parameters *f*_1_, *f*_2_, *f*_3_ are calculated according to (12). The final values are *f*_1_ = 1.143, *f*_2_ = 1.396, *f*_3_ = 1.142.
4	4th hidden layer	By applying the Smooth ReLU activation function to the linear combinations *f_i_*, the parameters *g*_1_, *g*_2_, *g*_3_ are calculated according to (13). The final values are *g*_1_ = 1.319, *g*_2_ = 1.609, *g*_3_ = 1.319.
5	Output layer	The accepted weight and bias matrices are $v=\left(\begin{array}{c} 0.4\\ 0.3\\ 0.3 \end{array}\right),$*c* = 0.5. The neural network output signal is calculated according to (14). The final value is *y* = 1.907.

**Table 12:** Data processing results from training samples using a traditional filtration method (author’s research).

Stage Number	Stage Name	Results
1	Summation of signals with their noise	The summation of signals with their noise is carried out in the same way as in the neural network method (Table 11). The final values are $n_{TC}^{\left(1\right)}=0.898$, $T_{G}^{*\left(1\right)}=0.875$, and $n_{FT}^{\left(1\right)}=0.880$, similar to *h*_1_ = 0.898, *h*_2_ = 0.875, and *h*_3_ = 0.880.
2	Dynamic compensation	Signals and interference are adjusted using coefficients for each parameter. For a median-recursive filter, according to [70], it is advisable to use the following coefficients: 0.8 for the *n_TC_* parameter, 1.2 for the $T_{G}^{*}$ parameter, and 0.9 for the *n_FT_* parameter. Then: $Corrected\left(n_{TC}\right)=0.8\cdot Clear\left(n_{TC}\right)+Noice\left(n_{TC}\right),$ $Corrected\left(T_{G}^{*}\right)=1.2\cdot Clear\left(T_{G}^{*}\right)+Noice\left(T_{G}^{*}\right),$ $Corrected\left(n_{FT}\right)=0.9\cdot Clear\left(n_{FT}\right)+Noice\left(n_{FT}\right),$ Total values $n_{TC}^{\left(2\right)}=0.932$, $T_{G}^{*\left(2\right)}=1.007$, and $n_{FT}^{\left(2\right)}=0.918$, similar to *z*_1_, *z*_2_, and *z*_3_.
3	Filtration 1st stage	Signals and interference are adjusted using coefficients for each parameter. For a median-recursive filter, according to [71], it is advisable to use the following coefficients: 0.75 for the *n_TC_* parameter, 1.05 for the $T_{G}^{*}$ parameter, and 0.85 for the *n_FT_* parameter. Then: $Filtered\left(n_{TC}\right)=0.75\cdot Corrected\left(n_{TC}\right),$ $Filtered\left(T_{G}^{*}\right)=1.05\cdot Corrected\left(T_{G}^{*}\right),$ $Filtered\left(n_{FT}\right)=0.85\cdot Corrected\left(n_{FT}\right),$ Total values $n_{TC}^{\left(3\right)}=0.699$, $T_{G}^{*\left(3\right)}=1.057$, and $n_{FT}^{\left(3\right)}=0.780$, similar to *f*_1_, *f*_2_, and *f*_3_.
4	Filtration 2nd stage	Signals and interference are adjusted using coefficients for each parameter. For a median-recursive filter, according to [72], it is advisable to use the following coefficients: 0.65 for the *n_TC_* parameter, 0.70 for the $T_{G}^{*}$ parameter, and 0.60 for the *n_FT_* parameter. Then: $Filtered\left(n_{TC}\right)=0.65\cdot Corrected\left(n_{TC}\right),$ $Filtered\left(T_{G}^{*}\right)=0.70\cdot Corrected\left(T_{G}^{*}\right),$ $Filtered\left(n_{FT}\right)=0.60\cdot Corrected\left(n_{FT}\right),$ Total values $n_{TC}^{\left(3\right)}=0.454$, $T_{G}^{*\left(3\right)}=0.740$, and $n_{FT}^{\left(3\right)}=0.507$, similar to *f*_1_, *f*_2_, and *f*_3_.
5	Final result	The output signal is calculated as: $y=n_{TC}^{\left(4\right)}+T_{G}^{*\left(4\right)}+n_{FT}^{\left(4\right)}=1.701.$

**Table 13:** The obtained data comparison results (author’s research).

Stage Number	Method Type	Stage Name	Output Variable	Value	Comparison Results
1	Neural network	1st hidden layer	*h*_1_	0.898	The results obtained in the neural network’s 1st hidden layer are identical to the results obtained using traditional filtering methods.
			*h*_2_	0.875	
			*h*_3_	0.880	
	Traditional filtration method	Summation of signals with their noise	$n_{TC}^{\left(1\right)}$	0.898	
			$T_{G}^{*\left(1\right)}$	0.875	
			$n_{FT}^{\left(1\right)}$	0.880	
2	Neural network	2nd hidden layer	*z*_1_	0.988	The results obtained in the neural network’s 2nd hidden layer are up to 44.1% higher than the results obtained using traditional filtering methods.
			*z*_2_	1.172	
			*z*_3_	0.984	
	Traditional filtration method	Dynamic compensation	$n_{TC}^{\left(2\right)}$	0.932	
			$T_{G}^{*\left(2\right)}$	1.007	
			$n_{FT}^{\left(2\right)}$	0.918	
3	Neural network	3rd hidden layer	*f*_1_	1.143	The results obtained in the neural network’s 3rd hidden layer are up to 44.1% higher than the results obtained using traditional filtering methods.
			*f*_2_	1.396	
			*f*_3_	1.142	
	Traditional filtration method	Filtration 1st stage	$n_{TC}^{\left(3\right)}$	0.699	
			$T_{G}^{*\left(3\right)}$	1.057	
			$n_{FT}^{\left(3\right)}$	0.780	
4	Neural network	4th hidden layer	*g*_1_	1.319	The results obtained in the neural network’s 4th hidden layer are up to 68.5% higher than the results obtained using traditional filtering methods.
			*g*_2_	1.609	
			*g*_3_	1.319	
	Traditional filtration method	Filtration 2nd stage	$n_{TC}^{\left(4\right)}$	0.454	
			$T_{G}^{*\left(4\right)}$	0.740	
			$n_{FT}^{\left(4\right)}$	0.507	
5	Neural network	Final result	*y*	1.907	The output signal value obtained in the neural network’s output layer is 10.8% higher than its value obtained using traditional filtering methods.
	Traditional filtration method	Final result	*y*	1.701	

**Table 14:** The neural network signal integration based on the filtering method 1st and 2nd types errors calculating results with traditional filters (author’s research).

Error Type	Neural Network Integration	Median Recursive Filter	Recursive Filter	Median Filter
Type I error, %	0.86	1.82	2.49	3.60
Type II error, %	0.38	0.80	1.10	1.59

## Data Availability

Data are contained within the article.
